# SARS-CoV-2 infection produces chronic pulmonary epithelial and immune cell dysfunction with fibrosis in mice

**DOI:** 10.1126/scitranslmed.abo5070

**Published:** 2022-07-07

**Authors:** Kenneth H. Dinnon, Sarah R. Leist, Kenichi Okuda, Hong Dang, Ethan J. Fritch, Kendra L. Gully, Gabriela De la Cruz, Mia D. Evangelista, Takanori Asakura, Rodney C. Gilmore, Padraig Hawkins, Satoko Nakano, Ande West, Alexandra Schäfer, Lisa E. Gralinski, Jamie L. Everman, Satria P. Sajuthi, Mark R. Zweigart, Stephanie Dong, Jennifer McBride, Michelle R. Cooley, Jesse B. Hines, Miriya K. Love, Steve D. Groshong, Alison VanSchoiack, Stefan J. Phelan, Yan Liang, Tyler Hether, Michael Leon, Ross E. Zumwalt, Lisa M. Barton, Eric J. Duval, Sanjay Mukhopadhyay, Edana Stroberg, Alain Borczuk, Leigh B. Thorne, Muthu K. Sakthivel, Yueh Z. Lee, James S. Hagood, Jason R. Mock, Max A. Seibold, Wanda K. O’Neal, Stephanie A. Montgomery, Richard C. Boucher, Ralph S. Baric

**Affiliations:** ^1^ Department of Microbiology & Immunology, University of North Carolina at Chapel Hill, Chapel Hill, North Carolina 27599, USA; ^2^ Department of Epidemiology, University of North Carolina at Chapel Hill, Chapel Hill, North Carolina 27599, USA; ^3^ Marsico Lung Institute, University of North Carolina at Chapel Hill, Chapel Hill, North Carolina 27599, USA; ^4^ Lineberger Comprehensive Cancer Center, University of North Carolina at Chapel Hill, Chapel Hill, North Carolina 27599, USA; ^5^ Center for Genes, Environment, and Health, National Jewish Health, Denver, Colorado 80206, USA; ^6^ Golden Point Scientific Laboratories, Hoover, Alabama 35216, USA; ^7^ Division of Pathology, Department of Medicine, National Jewish Health, Denver, Colorado 80206, USA; ^8^ NanoString Technologies, Seattle, Washington 98109, USA; ^9^ Department of Pathology and Laboratory Medicine, Mayo Clinic, Rochester, Minnesota 55905, USA; ^10^ Office of the Chief Medical Examiner, Oklahoma City, Oklahoma 73105, USA; ^11^ Department of Pathology, Cleveland Clinic, Cleveland, Ohio 44195, USA; ^12^ Weill Cornell Medicine, New York, New York 10065, USA; ^13^ Department of Pathology and Laboratory Medicine, University of North Carolina at Chapel Hill, Chapel Hill, North Carolina 27599, USA; ^14^ Department of Radiology, University of North Carolina at Chapel Hill, North Carolina 27599, USA; ^15^ Biomedical Research Imaging Center, University of North Carolina at Chapel Hill, Chapel Hill, North Carolina 27599, USA; ^16^ Department of Pediatrics, Pulmonology Division and Program for Rare and Interstitial Lung Disease, University of North Carolina at Chapel Hill, Chapel Hill, North Carolina 27599, USA; ^17^ Division of Pulmonary Diseases and Critical Care Medicine, University of North Carolina at Chapel Hill, Chapel Hill, North Carolina 27599, USA; ^18^ Department of Pediatrics, National Jewish Health, Denver, Colorado 80206, USA; ^19^ Division of Pulmonary Sciences and Critical Care Medicine, Department of Medicine, University of Colorado-Anschutz Medical Campus, Aurora, Colorado 80045, USA; ^20^ Rapidly Emerging Antiviral Drug Discovery Initiative, University of North Carolina at Chapel Hill, Chapel Hill, North Carolina 27599, USA

## Abstract

A subset of individuals who recover from coronavirus disease 2019 (COVID-19) develop post-acute sequelae of SARS-CoV-2 (PASC), but the mechanistic basis of PASC-associated lung abnormalities suffers from a lack of longitudinal tissue samples. The mouse-adapted severe acute respiratory syndrome coronavirus 2 (SARS-CoV-2) strain MA10 produces an acute respiratory distress syndrome (ARDS) in mice similar to humans. To investigate PASC pathogenesis, studies of MA10-infected mice were extended from acute to clinical recovery phases. At 15 to 120 days post-virus clearance, pulmonary histologic findings included subpleural lesions composed of collagen, proliferative fibroblasts, and chronic inflammation, including tertiary lymphoid structures. Longitudinal spatial transcriptional profiling identified global reparative and fibrotic pathways dysregulated in diseased regions, similar to human COVID-19. Populations of alveolar intermediate cells, coupled with focal up-regulation of pro-fibrotic markers, were identified in persistently diseased regions. Early intervention with antiviral EIDD-2801 reduced chronic disease, and early anti-fibrotic agent (nintedanib) intervention modified early disease severity. This murine model provides opportunities to identify pathways associated with persistent SARS-CoV-2 pulmonary disease and test countermeasures to ameliorate PASC.

## Teaser

After recovery from acute SARS-CoV-2 infection, mice exhibit chronic lung disease similar to some humans, allowing for testing of therapeutics.

## INTRODUCTION

The ongoing coronavirus disease 2019 (COVID-19) pandemic is caused by the severe acute respiratory syndrome coronavirus 2 (SARS-CoV-2) (*1*, *2*). New antivirals, antibody therapies, vaccinations, and improved critical care strategies have diminished acute fatality rates (*3*). However, about 40% of symptomatic and asymptomatic COVID-19 survivors develop post-acute sequelae, termed PASC or “long-COVID”, with features that include dyspnea, fatigue, chest pain, cognitive decline, and multi-organ damage, especially chronic lung disease (*4*–*9*). Models are urgently needed to discover early biomarkers and countermeasures to identify and prevent PASC.

COVID-19 is generally characterized as biphasic, with an acute phase dominated by active SARS-CoV-2 infection and a post-viral clearance phase dominated by host reparative and immunologic processes (*10*). Human autopsy samples highlight the lung disease manifestations in patients who succumbed to COVID-19 (*11*, *12*), with broad features of chronic active pneumonia (CAP), alveolar architectural destruction, dense cellularity, and pulmonary fibrosis (PF) with myofibroblast proliferation and collagen deposition (*13*–*19*). Survivors of previous emerging coronavirus infections reported severe post-infectious fibrotic lung sequelae long after virus clearance, and autopsy data suggest similar late sequelae will follow SARS-CoV-2 infections (*20*–*26*). However, elucidating the pathogenesis of post-SARS-CoV-2 lung disease is difficult because autopsy samples describe disease at single time points and are highly heterogeneous. Moreover, mechanisms describing the development of non-viral CAP or PF in humans are poorly understood, providing only partial roadmaps on which to base studies of SARS-CoV-2 pulmonary pathogenesis (*27*). Animal models offer opportunities to fill these knowledge gaps. Although PASC is a heterogeneous multi-organ system condition in humans, respiratory sequelae are among the most prominent. Thus, we have focused this study on understanding the chronic active inflammatory and fibrotic sequelae following SARS-CoV-2 in an animal model.

SARS-CoV-2 infection models in standard laboratory mice that produce ARDS and phenocopy age-related acute SARS-CoV-2 disease are available (*28*), but PASC-like disease phenotypes in the lung after virus clearance have not been reported. We characterized the spatial and temporal patterns associated with the long-term (120 day) pulmonary consequences of SARS-CoV-2 mouse-adapted strain (MA10) infection in standard BALB/c laboratory mice (*28*). Lung disease in mice surviving acute SARS-CoV-2 MA10 infection was investigated using complementary virologic, histologic, and immunologic techniques supplemented with immunohistochemistry (IHC) and CT scanning. Digital spatial profiling (DSP) and RNA in situ hybridization (ISH) were utilized to identify transcriptional profiles during acute and chronic disease phases to characterize tissue damage and repair in mice and humans. Countermeasures to prevent lung disease sequelae for SARS-CoV-2 infection were investigated.

## RESULTS

### SARS-CoV-2 MA10 infection produces chronic pulmonary disease.

PASC outcomes were investigated in young (10-week-old) and more susceptible aged (1-year-old) mice through 120 days post infection (dpi) (*28*). To induce severe acute disease without excessive mortality, 1-year-old female BALB/c mice were inoculated intranasally with 10^3^ plaque-forming units (PFU) of SARS-CoV-2 MA10 (*28*). Young female mice received 10^4^ PFU to achieve similar disease severity during the acute phase including peak lung titers (about 10^7^ PFU) at 2 dpi. Reflecting recent recommendations for diagnosing Acute, Ongoing and Post-COVID-19 syndrome in humans (including chronic signs or symptoms after 12 weeks) (*29*), mice were necropsied at 2, 7, 15, 30, 60, and 120 dpi to measure lung viral titers and for additional analyses.

**Fig. 1.**
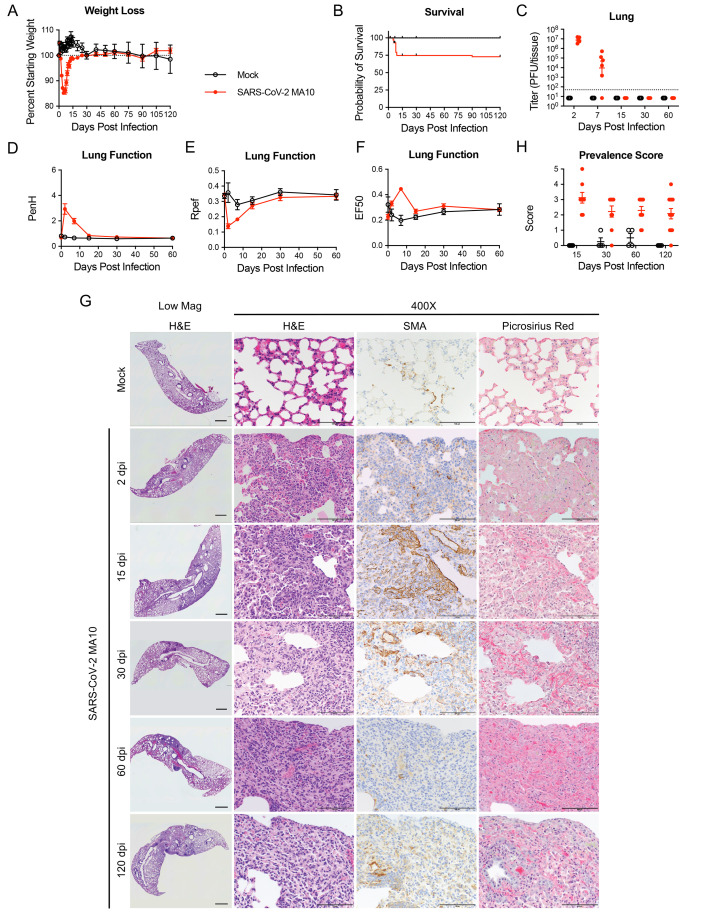
SARS-CoV-2 MA10 infection causes lung damage in aged surviving mice. 1-year-old female BALB/c mice were infected with 10^3^ PFU SARS-CoV-2 MA10 (n=74) or PBS (n=24) and monitored for **(A)** percent starting weight and **(B)** survival. **(C)** Log transformed infectious virus lung titers were assayed at indicated time points. Dotted line represents LOD. Undetected samples are plotted at half the LOD. **(D to F)** Lung function was assessed by whole body plethysmography for **(D)** PenH, **(E)** Rpef, and **(F)** EF50. **(G)** Histopathological analysis of lungs at indicated time points are shown. H&E indicates hematoxylin and eosin staining. SMA indicates DAB-labeling (brown) immunohistochemistry for α-smooth muscle actin. Picrosirius Red staining (bright pink-red) highlights collagen fibers. Image scale bars represents 1000 μm for low magnification and 100 μm for 400X images. **(H)** Disease incidence scoring is shown for indicated time points: 0 = 0% of total area of examined section, 1 = less than 5%; 2 = 6 to 10%; 3 = 11 to 50%; 4 = 51 to 95%; 5 = greater than 95%. Graphs represent individuals necropsied at each time point **(C and H)**, with the average value for each treatment and error bars representing standard error of the mean. Mock infected animals represented by open black circles and SARS-CoV-2 MA10 infected animals are represented by closed red circles.

Replicating previous findings (*28*), acute infection in 1-year-old mice resulted in rapid decreases in body weight and 25% mortality over 7 days compared to controls (**Fig. 1A and B**). Surviving aged mice cleared culturable infection by 15 dpi, restored lung function by 15 dpi, and recovered body weight by 30 to 60 dpi (100% starting weight) (**Fig. 1C to F**). Features of acute (2 to 7 dpi) lung injury following SARS-CoV-2 MA10 infection in 1-year-old mice included heterogeneous inflammation and alveolar damage with consolidation, edema, fibrin and protein exudates, and occasional hyaline membranes (**Fig. 1G**) (*28*). By 15 through 120 dpi, a high incidence of histologically heterogeneous lung disease was observed (**Fig. 1G and H**). The distribution of diseased areas remained relatively constant over the 15 to 120 dpi interval, suggesting disease developed focally early and persisted. Diseased regions were often subpleurally oriented and characterized by dense hypercellularity composed of admixed immune cell accumulation (often organized into tertiary lymphoid structures), abundant smooth muscle actin (SMA) positive fibroblasts (myofibroblasts), and collagen deposition as detected by Picrosirius Red staining of collagen fibers, characteristic of CAP and PF. Micro-CT scanning of 15 and 30 dpi 1-year-old mice identified dense subpleural opacities (**fig. S1A**; 10-week-old animals **fig. S1B**), and lack of honeycombing, similar to the mouse histologic lesions (**Fig. 1G and H**) and human fibrotic lung disease (*30*, *31*).

Chronic manifestations were not limited to susceptible 1-year-old BALB/c mice. MA10 infection (10^4^ PFU) in 10-week BALB/c mice also caused acute weight loss (**fig. S2A**) and 25% mortality (**fig. S2B**). Although young mice cleared infectious virus earlier than old mice (by 7 dpi, **fig. S2C**), transient pulmonary dysfunction was still observed (**fig. S2D to F**). Young mice exhibited subpleural lesions similar to old mice at 15 and 30 dpi, but the severity of disease usually diminished over 120 dpi, suggesting young mice may have a higher capacity for repair (**fig. S2G and H**). Of note, about 20% of young animals evaluated on days 30, 60 and 120 had maximal CAP/fibrotic lesion disease prevalence scores, whereas 40% returned to baseline, reflecting heterogeneity seen in human patients. Importantly, chronic disease was not unique to BALB/c mice. SARS-CoV-2 MA10 infection (10^4^ PFU) in 1-year-old female C56BL/6J mice caused acute weight loss (**fig. S3A**) and 30% mortality (**fig. S3B**), with viral clearance by 15 dpi (**fig. S3C**). Fibrotic lesion incidence peaked at 15 dpi (**fig. S3D and E**) with scores similar to 1-year-old BALB/c mice (**Fig. 1H**). Fibrotic lesions were reduced by 30 dpi in C57BL/6J mice, with 50% still containing fibrotic disease. In summary, CAP/fibrotic lesions were observed in female mice of multiple mouse strains and ages. Because the most severe and persistent pulmonary lesions were observed in 1-year-old BALB/c mice, this strain and age was selected for more intensive studies of PASC pathogenesis in mice.

### Aged mice exhibit sustained lung and blood cytokine responses to SARS-CoV-2 lung infection.

Cytokine analysis of lung homogenate and serum samples from both age groups revealed robust cytokine responses to infection (**fig. S4A and B, data files S1 and S2**). Lung cytokine responses were generally more pronounced at 2 dpi in young mice who received higher inocula. However, old mice exhibited more sustained responses post 7 dpi (**fig. S4A**). In particular, C-X-C motif chemokine ligand 5 (CXCL5), macrophage colony-stimulating factor (M-CSF), interleukin (IL)-19, and IL-33, which enhance pro-fibrogenic type 2 cytokine production in a macrophage-dependent manner (*32*), remained persistently elevated in lungs to 30 dpi in older, but not younger, mice. In serum, a similar pattern of more robust cytokine response in young versus old mice 2 dpi was observed (**fig. S4B**). Antiviral interferons (IFN-α and IFN-λ1) were highly expressed at 2 dpi and returned to baseline by 7 dpi at both ages (**fig. S4A**). The more robust acute lung and plasma cytokine responses in younger versus older mice were associated with more rapid viral clearance in younger mice (by 7 dpi) (**fig. S2C and S4**). The persistently elevated lung cytokine responses in older mice after 7 dpi may reflect delayed virus clearance or defective reparative capacity (*33*, *34*).

### SARS-CoV-2 MA10 infection produces acute and chronic inflammatory cell responses.

Immunoinflammatory cellular responses to SARS-CoV-2 MA10 infection and injury included recruitment of macrophages, T cells, and B cells (**fig. S5**) (*35*). Lymphoid aggregates identified in dense cellular regions at 15 to 120 dpi consisted of a spectrum of lymphocyte subsets, including CD4^+^ and CD8^+^ T cells as well as B cells (**fig. S5A and B**). Immunohistochemistry (IHC) was used to quantitate the kinetics of CD4^+^ and CD8^+^ T cells (**fig. S5C and D).** Increased CD4^+^ T cells appeared as early as 2 dpi, peaked at 7 to 15 dpi, and persisted through 120 dpi (**fig. S5A**). CD8^+^ T cell accumulation peaked at 15 dpi and remained at lower frequencies through 120 dpi (**fig. S5A and D**). B220^+^ B cell accumulation was observed at 7 dpi and sustained thereafter. CD68^+^ macrophages were increased at 7 dpi and remained elevated at 120 dpi in dense cellular regions, whereas inducible nitric oxide synthase (iNOS)^+^ M1 and Arginase^+^ M2 macrophages peaked at 2 and 7 dpi, respectively, and remained elevated at lower frequencies thereafter, suggesting involvement of multiple subsets of macrophages in inflammatory and reparative process with different kinetics.

Flow cytometry at 30 dpi revealed that total cells, CD45^+^ immune, and CD31^+^ endothelial cells were increased (**fig. S5E and F**), consistent with IHC results (**fig. S5A and B**). CD4^+^ T cells and CD19^+^ B cells were increased in infected mice (**fig. S5G**), consistent with prolonged inflammatory immune responses in pulmonary fibrotic diseases (*36*). Within the monocyte/macrophage lineage, interstitial macrophages were elevated in infected mice at 30 dpi (**fig. S5H**), consistent with a documented role that macrophages play in lung remodeling in pulmonary fibrosis (*37*).

### Host transcriptional profiles are spatially and temporally altered in response to SARS-CoV-2 infection.

**Fig. 2.**
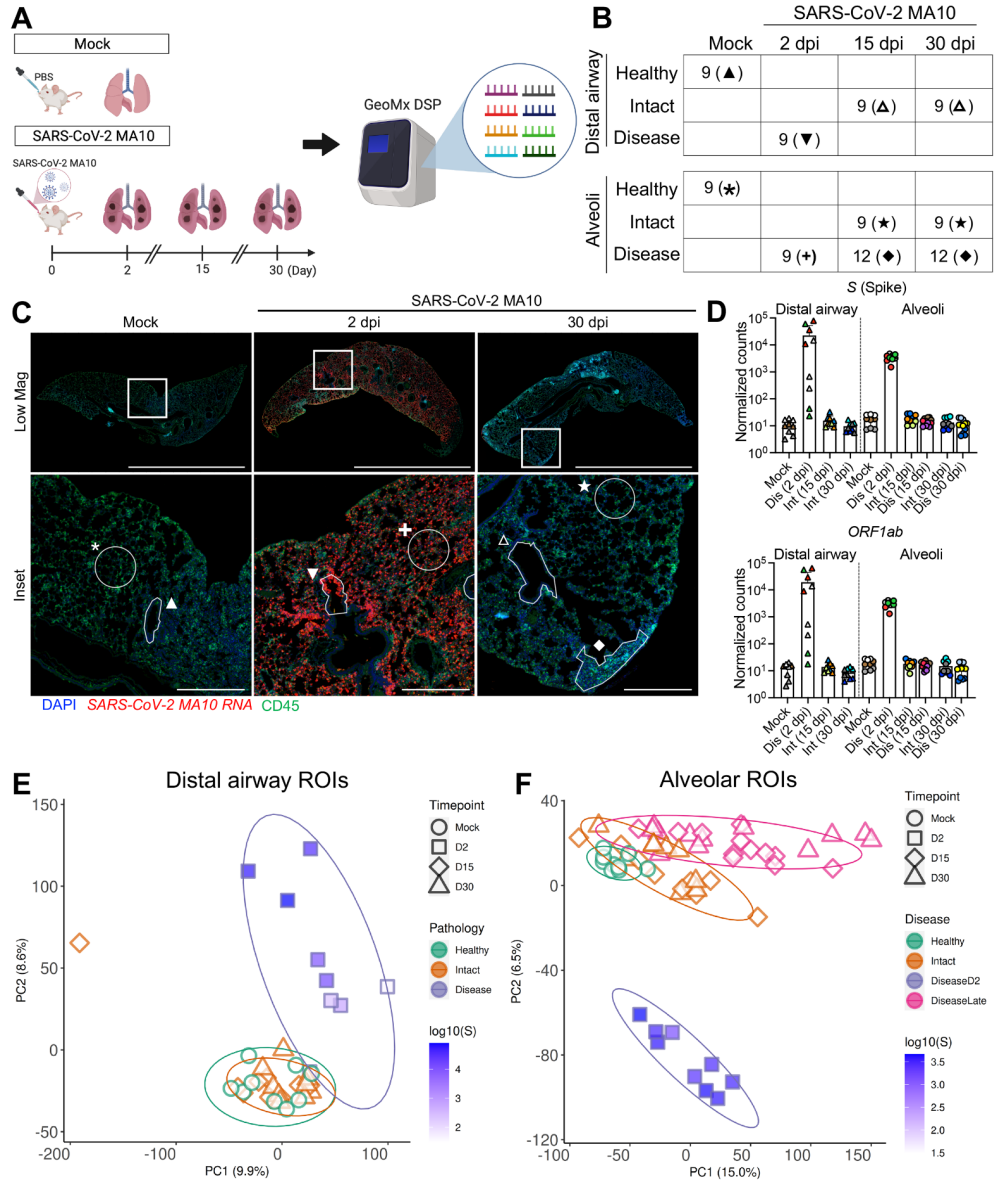
**Transcriptional digital spatial profiling reveals unique signatures in diseased tissue compartments. (A)** Experimental setup for GeoMx digital spatial profiling (DSP). Regions of interest (ROIs) were selected from formalin-fixed paraffin embedded tissue sections from mock, 2, 15, and 30 dpi 1-year-old female BALB/c mice and analyzed by NanoString GeoMx Whole Transcriptome Atlas. **(B)** A table summarizing ROIs from each tissue compartment, disease state, and time point is shown. Each time point includes 3 independent mouse samples, with 3 to 4 ROIs per mouse. **(C)** Example of ROI selections from mock, 2 dpi, and 30 dpi post SARS-CoV-2 MA10 lungs are shown. Scale Bars = 5 mm for low magnification images and 500 μm for insets. Symbols define representative ROIs for each tissue compartment, disease state, and time point as indicated in the table in panel (**B**). Tissue sections were stained for nuclei (DAPI: blue), SARS-CoV-2 MA10 RNA (red), and CD45 (green). **(D)** DSP Q3 normalized counts of SARS-CoV-2 MA10 Spike (*S*) and *ORF1ab* expression are shown for mock, infected diseased (Dis), or intact (Int) ROIs. Graphs represent all ROIs selected with each unique color representing one animal, bars represent average value of each group with error bars representing standard error of the mean. **(E and F)** PCA plots of distal airway **(E)** and alveolar **(F)** ROIs are shown. Graphs represent all ROIs selected with each unique color and symbol representing disease state and time point, respectively. DSP Q3 normalized counts of SARS-CoV-2 MA10 Spike (*S*) of ROIs are represented by purple-blue fill color intensity.

GeoMx digital spatial profiling (DSP) was employed to interrogate viral and mouse RNA expression in pulmonary lesions from a subset of mock versus infected 1-year-old mice at 2, 15, and 30 dpi (**Fig. 2A**). GeoMx DSP allows for quantitative analyses of RNA transcripts within targeted regions of interest (ROIs) using barcoded antisense oligos hybridized to over 19,000 host and viral transcripts on formalin fixed paraffin embedded tissue sections. Since SARS-CoV-2 MA10 primarily infects alveolar type II (AT2) cells and terminal bronchiolar secretory club cells (*28*), we focused on these two regions. At 2 dpi, alveolar ROIs were selected based on the presence of SARS-CoV-2 MA10 RNA positive cells. Bronchiolar ROIs at 2 dpi were selected to represent a range of SARS-CoV-2 MA10 infection. At later time points (15, 30 dpi), the heterogeneity of alveolar lung infection/responses was sampled by obtaining ROIs from morphologically “diseased” regions with hypercellularity versus morphologically “intact” regions. All distal airways appeared normal at 15 and 30 dpi with ROIs defined as “intact”. Following data quality control and normalization, 60 alveolar and 36 bronchiolar epithelial ROIs from SARS-CoV-2 MA10-infected or mock mice were sampled at acute (2 dpi) and late (15 and 30 dpi) time points (**Fig. 2B and C** **, data file S3**). Quantification of viral RNAs demonstrated clearance of viral RNAs from intact and diseased alveolar ROIs by 15 dpi (**Fig. 2D**), concordant with clearance of infectious virus (**Fig. 1C**).

Principal component analysis (PCA) of expressed genes identified time, region, and virus-dependent effects (**Fig. 2E and F**). High virus transcript positive regions at 2 dpi clustered away from mock in both distal airway and alveolar regions. Further, the alveolar ROIs selected from diseased regions of infected mice at 15 and 30 dpi separated from mock, suggesting persistent alterations of host transcriptomes (**Fig. 2F**). In contrast, the ROIs selected from “intact” airway and alveolar regions at 15 and 30 dpi clustered near mock healthy ROIs, suggesting recovery (**Fig. 2E and F**).

**Fig. 3.**
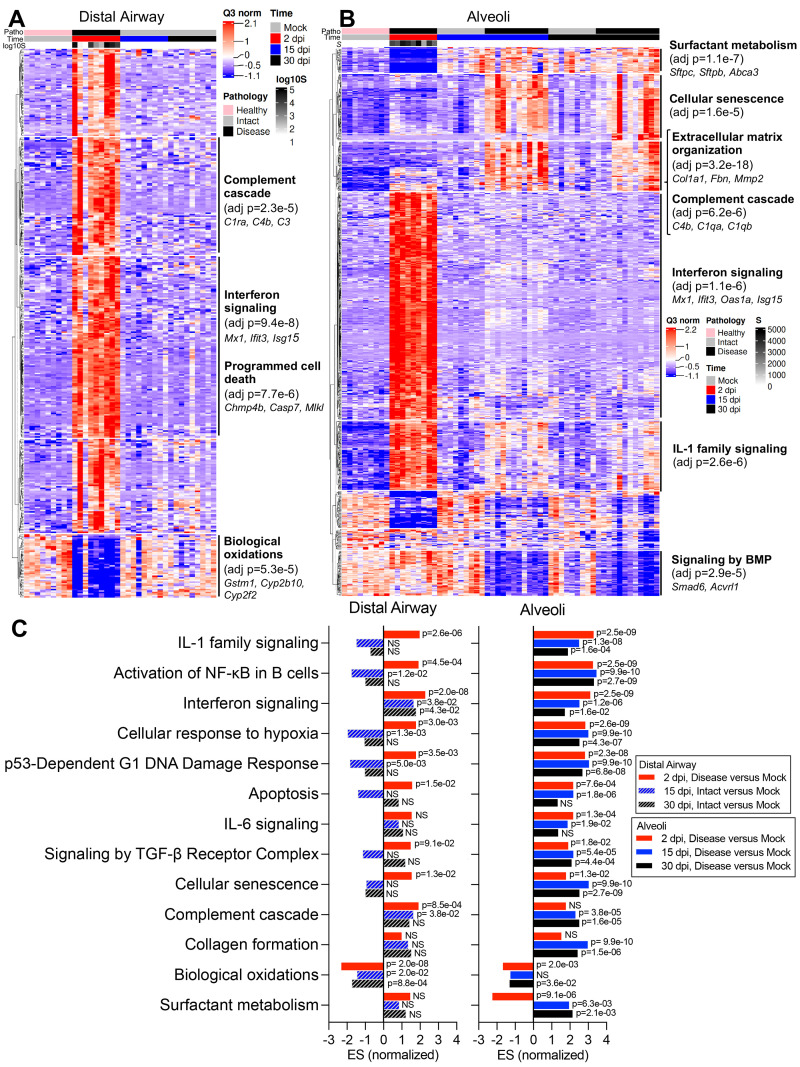
**Digital spatial profiling reveals distinct transcriptional pathway changes during acute and late stages of SARS-CoV-2 disease. (A and B)** DSP heatmaps are shown for differentially expressed genes (DEGs) in ROIs across all time points in **(A)** distal airway and **(B)** alveolar tissue compartments. DEGs were obtained by comparing DSP Q3 normalized counts of transcripts between ROIs at 2, 15, and 30 dpi versus mock-infected 1-year-old female BALB/c mice. **(C)** Normalized enrichment scores (ES) and adjusted p-values obtained by DSP pathway enrichment analysis are shown for distal airway and alveolar ROIs at 2, 15, or 30 dpi versus mock. Statistical analyses used are detailed in methods. NS, not significant.

Consistent with PCA, viral infection induced major changes in transcriptome profiles in infected mouse lungs (**Fig. 3A and B** **; data files S2 through S4**). In both alveoli and bronchioles, virally infected disease ROIs at 2 dpi were characterized by a broad and robust up-regulation of viral infection-induced acute inflammatory genes, represented by enrichment of IFN, IL-1, and nuclear factor (NF)-κB signaling pathways (**Fig. 3A to C** **, data files S2 and S5**). Up-regulated IFN-stimulated genes (ISGs) were consistent with ISGs reported in human cells after emerging coronavirus infection (**fig. S6A to C; data file S2**) (*38*, *39*), suggesting common antiviral pathways are activated in human and mouse pulmonary cells. As noted in other human lung cell types after coronavirus infection (*40*), ISG expression patterns in airway and alveolar ROIs were not identical, with some ISGs more robustly up-regulated in airway epithelial (*Ifitm1*, *Lap3*, *Epsti1*) (**fig. S6C and D**) or alveolar ROIs (*Ifitm2*, *Batf2*, *Samhd1*) (**fig. S6C and E**). By 15 and 30 dpi, the expression of most ISGs returned to degrees similar to mock infection (**Fig. 1C** **,** **2D** **,** **3A and B** **, fig. S6C**).

DSP pathway analyses revealed down-regulation of biological oxidation (bronchiolar and alveoli) and surfactant metabolism (alveoli) in infected mice at 2 dpi (**Fig. 3A and B**), associated with loss of secretory club (*Cyp2f2, Scgb1a1*, *Scgb3a2*) and AT2 (*Sftpc, Lamp3, Abca3*) cell markers (**fig. S7A**). RNA-ISH confirmed that SARS-CoV-2 MA10 RNA was predominantly localized in *Scgb1a1*+ secretory club cells and *Sftpc*+ AT2 cells at 1 dpi in bronchioles and alveoli, respectively (**fig. S7B and C**). Loss of club (*Scgb1a1*) and AT2 (*Sftpc*) cell marker expression accompanied SARS-CoV-2 MA10 infection at 1 and 2 dpi, followed by restoration to baseline values by 15 dpi (**fig. S7A to E**). The early loss of *Scgb1a1* and surfactant protein genes is consistent with reported human COVID-19 autopsy data (*41*). Ciliated (*Foxj1, Dnah5, Rsph1*) and AT1 (*Ager, Hopx, Cav1*) cell markers were minimally affected by MA10 infection at any time point (**fig. S7A to C and F**).

The transcriptomic analyses also revealed striking temporal differences in gene expression in alveolar versus bronchiolar regions (**Fig. 3A to C**). Consistent with failure of “diseased” alveolar regions to return to histologically “intact”-like states, pathway analyses at 30 dpi revealed persistently up-regulated cellular senescence, hypoxia signaling, complement activation, P53 damage responses, signaling by the transforming growth factor (TGF)-β receptor complex, collagen formation, and extracellular matrix organization pathways, unique to diseased alveolar regions. The difference in post-infection recovery between the bronchiolar (rapid, complete) versus alveolar regions (slow, incomplete) was also notable. Because apoptosis is reported to be less inflammatory than necrotic cell death (*42*), we investigated whether apoptotic cellular responses to infection were different between the two regions (**fig. S7G)**. At 2 dpi, SARS-CoV-2 MA10-infected bronchiolar epithelial cells expressed evidence of activated apoptotic pathways (cleaved caspase-3). In contrast, alveolar regions were characterized by widespread infection but little cleaved caspase-3. These differences in apoptotic activity are consistent with reports that murine airway epithelial cells are more primed for apoptosis than alveolar epithelial cells in basal states (*43*).

### Transcriptional digital spatial profiling reveals alveolar epithelial damage and regeneration following SARS-CoV-2 infection in mice.

Recent single-cell RNA sequencing studies in acute alveolar injury mouse models have identified unique AT2 to AT1 transitional alveolar epithelial cell types following alveolar damage (*44*–*46*). These cells are defined variably as a Krt8+ alveolar differentiation intermediate (ADI) (*44*), damage-associated transient progenitor (DATP) (*45*), or pre-AT1 transitional state cell (PATS) (*46*) (ADI/DATP/PATS hereafter). Incomplete transition from AT2 to AT1 cells, with an accumulation of ADI/DATP/PATS cells, has also been identified in human idiopathic pulmonary fibrosis (IPF) (*46*) and in COVID-19 postmortem lungs (*47*, *48*), suggesting a common dysfunction in prolonged epithelial repair/disrepair.

**Fig. 4.**
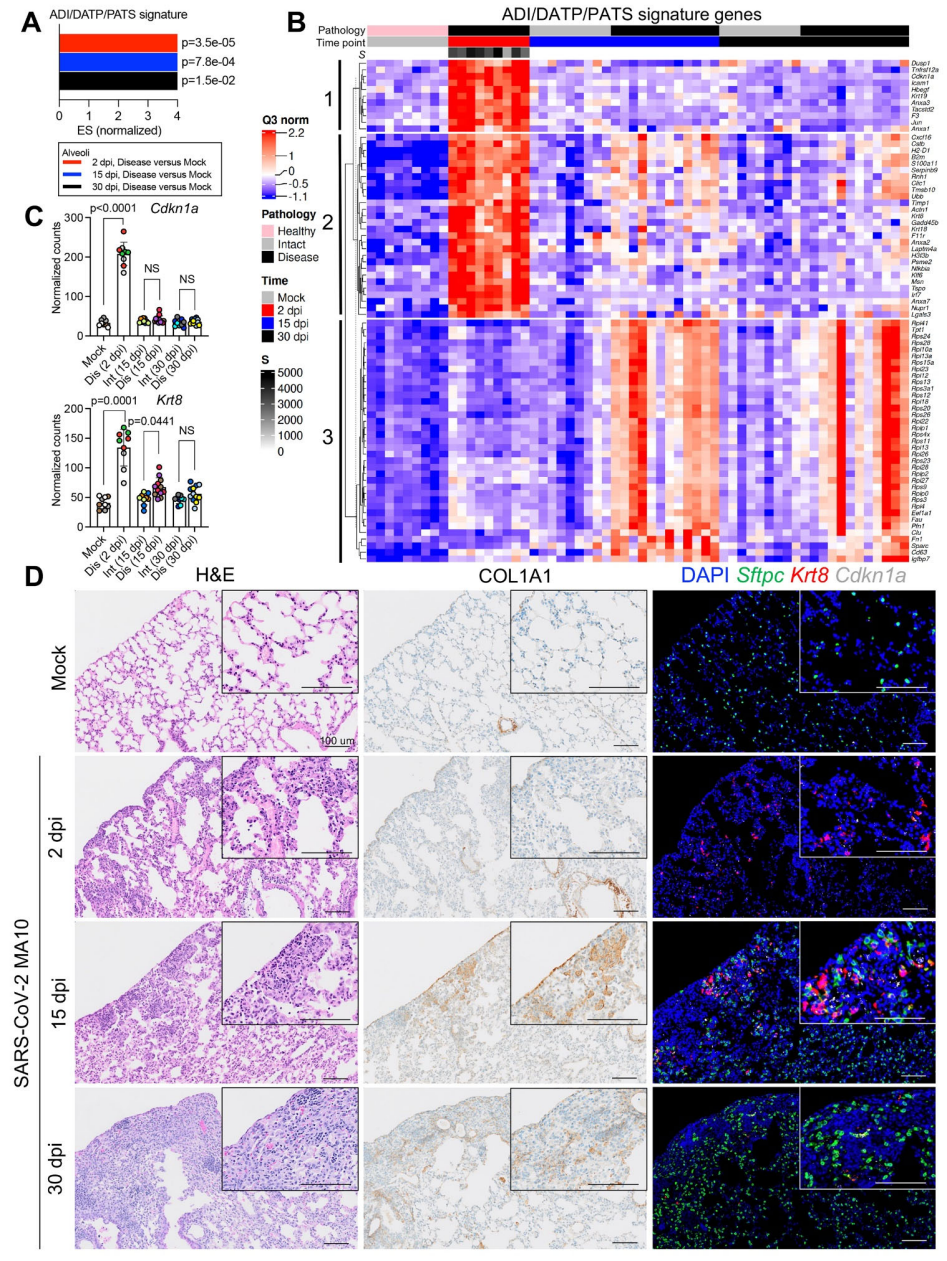
**Transitional alveolar epithelial cell genes are up-regulated following SARS-CoV-2 MA10 infection. (A)** Normalized enrichment scores (ES) and adjusted p-values are shown for a customized ADI/DATP/PATS signature in diseased alveolar ROIs at 2, 15, or 30 dpi versus mock 1-year-old female BALB/c mice. The customized ADI/DATP/PATS signature was obtained from a reported data set (*44*–*46*). **(B)** A DSP heatmap of reported ADI/DATP/PATS marker genes in alveolar ROIs is shown for mock, 2, 15, and 30 dpi 1-year-old female BALB/c mice. DSP Q3 normalized counts of SARS-CoV-2 MA10 Spike (*S*) of ROIs are log10 transformed and represented by gray color intensity. **(C)** DSP Q3 normalized counts of *Cdkn1a* and *Krt8* expression across alveolar ROIs in mock, infected diseased (Dis), or intact (Int) ROIs at indicated time points in 1-year-old female BALB/c mice. Graphs represent all ROIs selected with each unique color representing one animal, bars represent average value of each group with error bars representing standard error of the mean. The difference in DSP Q3 normalized counts for targeted genes in ROIs between each condition and time point was statistically tested using a linear mixed-effect model with condition and time point as fixed effects and replicate mice as random-effect factors. NS, not significant. **(D)** Histopathological analysis is shown for lungs isolated from mock or SARS-CoV-2 MA10-infected 1-year-old female BALB/c mice at indicated time points. Left: hematoxylin and eosin staining. Middle: DAB-labeling (brown) immunohistochemistry for Col1a1. Right: RNA-ISH for *Sftpc*, *Krt8* and *Cdkn1a*. Scale Bars indicate 100 μm.

**Fig. 5.**
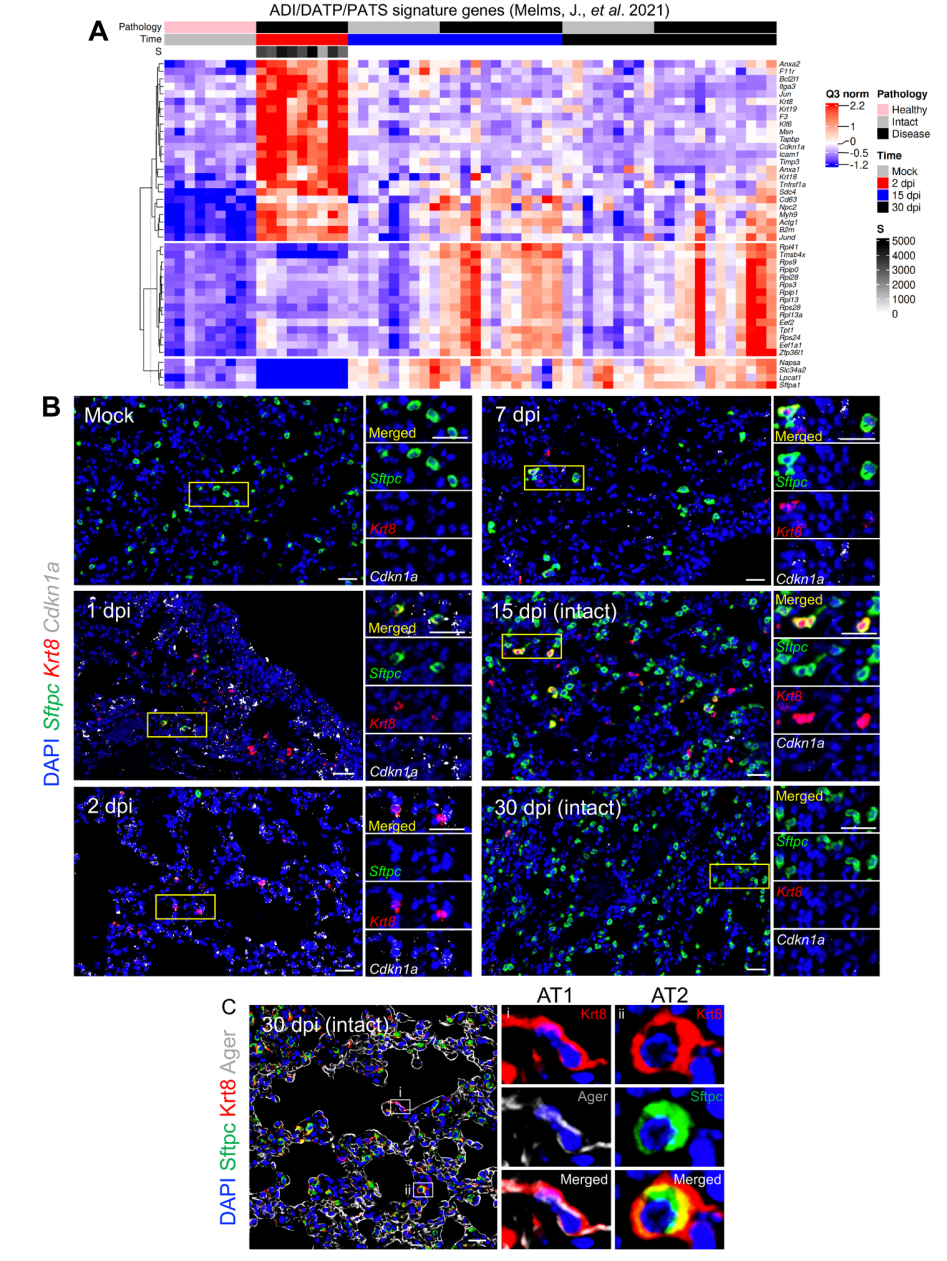
**Histological and transcriptional analyses reveal ADI/DATP/PATS cells are dynamically involved in alveolar regeneration following SARS-CoV-2 MA10 infection. (A)** A DSP heatmap is shown for of ADI/DATP/PATS signature genes identified in COVID-19 autopsy lungs (*47*) in alveolar ROIs in mock, 2, 15, and 30 dpi 1-year-old female BALB/c mice. DSP Q3 normalized counts of SARS-CoV-2 MA10 Spike (*S*) of ROIs are represented by gray color intensity. **(B)** RNA-ISH of *Sftpc* and ADI/DATP/PATS cell markers *Krt8* and *Cdkn1a* is shown over time course after SARS-CoV-2 MA10 infection, with selected areas of interest at indicated time points. Morphologically intact alveolar regions were selected at 15 and 30 dpi. Scale Bars = 25 μm. **(C)** Immunohistochemistry of Krt8 with AT1 (Ager) (i) and AT2 (Sftpc) (ii) cell markers is shown. Scale Bars = 20 μm.

Utilizing ADI/DATP/PATS signature genes reported from mouse acute lung injury (ALI) models (*44*–*46*), the SARS-CoV-2 MA10 DSP data demonstrated enrichment of ADI/DATP/PATS signatures in diseased alveolar ROIs at 2, 15, and 30 dpi (**Fig. 4A**). The ADI/DATP/PATS signature genes were categorized into three expression clusters (**Fig. 4B** **, data file S2**). The first cluster (*Cdkn1a/F3/Timp1*) was enriched in diseased ROIs at 2 dpi and decreased after 15 dpi, suggesting these genes may play a role in AT2 cell trans-differentiation into ADI/DATP/PATS cells. The second cluster (*Krt8/Cxcl16/Cstb*) exhibited increased expression at 2 dpi through 30 dpi. The third gene cluster (*Clu*/*Eef1a1*), including a variety of ribosomal protein genes, exhibited increased expression at 15 dpi and later. To further characterize the relationships between ADI/DATP/PATS cells and disease, combined RNA-ISH and DSP analyses of reported transitional ADI/DATP/PATS cell markers (*Cdkn1a*, *Krt8*) (*47*, *48*) were serially performed post infection (**Fig. 4C and D** **)**. DSP data demonstrated that: 1) *Cdkn1a* was up-regulated at 2 dpi and waned at late time points; and 2) *Krt8* was also up-regulated at 2 dpi and in diseased ROIs at 15 dpi (**Fig. 4C**). Although *Krt8*+/*Cdkn1a*+ RNA-ISH signals were not detectable in alveolar regions in mock mice, increased numbers of dual *Krt8+* and *Cdkn1a+* cells was observed by RNA-ISH in SARS-CoV-2-infected alveolar regions at 1 and 2 dpi (**Fig. 4D**), consistent with the DSP data (**Fig. 4B and C**). The murine DSP gene signatures exhibited features similar to ADI/DATP/PATS signature genes identified in human COVID-19 autopsy lungs (*47*) (**Fig. 5A** **, data file S2**), including p53, apoptosis, and hypoxia pathways (**Fig. 3B, C**).

*Sftpc*+ AT2 cells remaining in infected alveolar regions at 1 dpi co-expressed *Krt8* and *Cdkn1a* (**Fig. 5B**), consistent with the reported AT2 to ADI/DATP/PATS transitions after ALI in mice (*44*–*46*). At 2 dpi, *Krt8*+/*Cdkn1a*+ cells were present and *Sftpc*+/*Krt8*+ cells were rare (**Fig. 4D**), consistent with the loss of *Sftpc* in disease ROIs at 2 dpi (**fig. S7D and E**). At 7 to 15 dpi, *Sftpc* expression was restored and only occasional *Sftpc*+/*Krt8*+ cells were observed in repairing regions (**Fig. 5B**). Given the decreased viral titer (**Fig. 1C**) and restoration of *Sftpc* expression at 7 to 15 dpi (**fig. S7D and G**), *Sftpc*+/*Krt8*+ cells observed in these repairing regions likely reflected *Krt8*+ ADI/DATP/PATS cells re-transitioning into mature alveolar cells. Consistent with this notion, immunohistochemistry revealed co-expression of Krt8 with both AT1 (Ager) and AT2 (Sftpc) cell markers at 30 dpi (**Fig. 5C**). However, although *Sftpc*+ AT2 cells were restored in most alveolar regions at 15 to 30 dpi (**Fig. 4D** **, fig. S7C and D**), persistent *Krt8*+ or *Cdkn1a*+ cell clusters, coupled with muted restoration of *Sftpc*+ cells, was identified in dense cellular subpleural fibrotic alveolar regions where collagen alpha-1(I) chain (COL1A1) protein accumulation coexisted (**Fig. 4D**).

### Persistent inflammation and fibrosis are a chronic manifestation in SARS-CoV-2 MA10-infected mice.

**Fig. 6.**
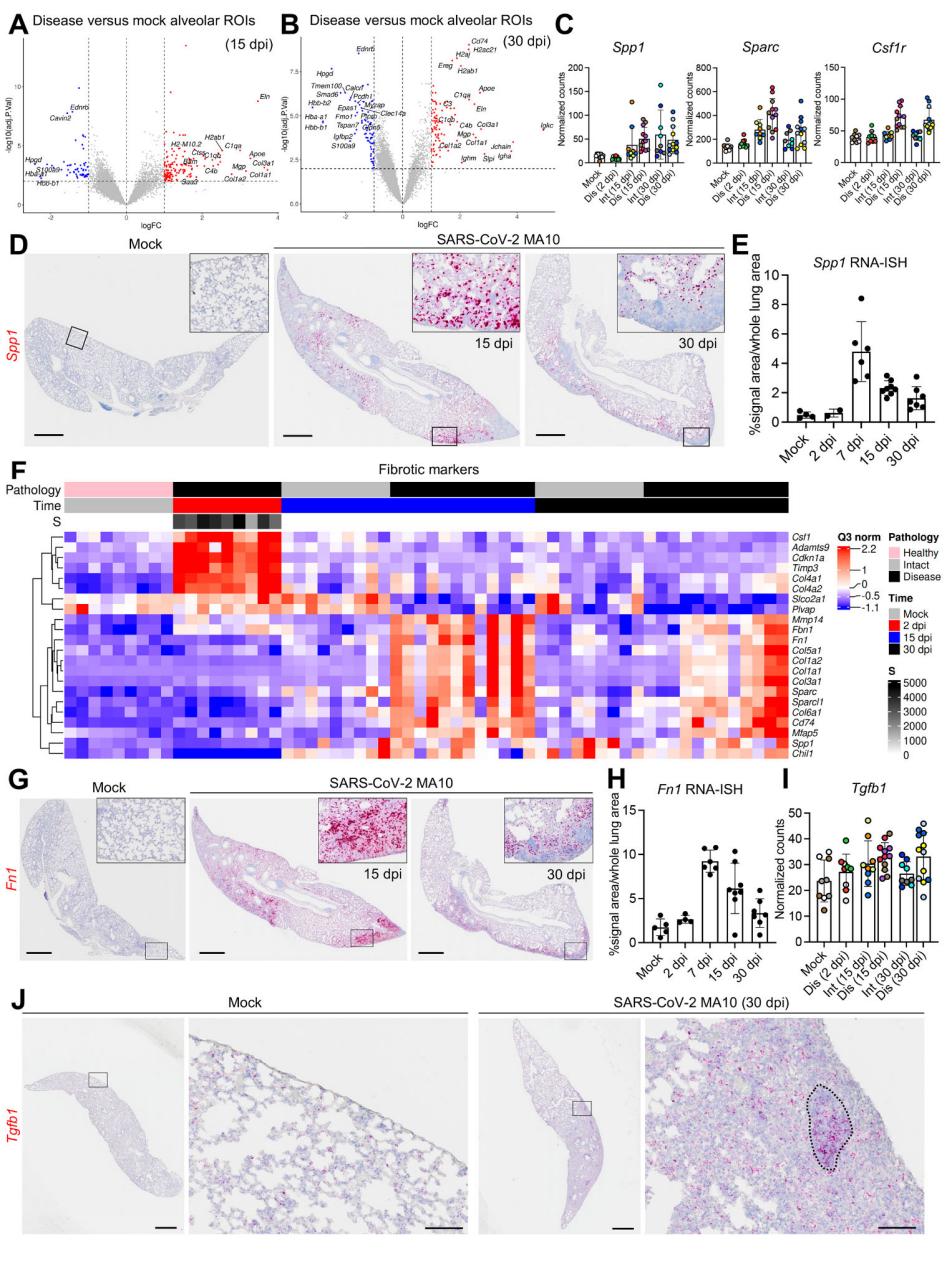
**SARS-CoV-2 MA10 infection induces profibrotic gene expression at late time points. (A and B)** Volcano plots of DSP DEGs are shown for diseased alveolar ROIs at **(A)** 15 and **(B)** 30 dpi versus mock 1-year-old female BALB/c mice. **(C)** DSP Q3 normalized counts of *Spp1*, *Sparc*, and *Csf1r* expression associated with profibrotic macrophage archetype are shown for mock, infected diseased (Dis), or intact (Int) ROIs at indicated time points in 1-year-old female BALB/c mice. **(D and E)** *Spp1* expression was measured by RNA-ISH **(D)** and quantified and normalized to whole lung area **(E)**. Scale bars indicate 1 mm. **(F)** DSP heatmap of selected profibrotic and fibrosis related genes in alveolar ROIs are shown for mock, 2, 15, and 30 dpi 1-year-old female BALB/c mice. DSP Q3 normalized counts of SARS-CoV-2 MA10 Spike (*S*) of ROIs are represented by gray color intensity. **(G and H)** *Fn1* expression (red) by RNA-ISH **(G)** with quantification **(H)** is shown. Scale bars indicate 1 mm. **(I)** DSP Q3 normalized counts of *Tgfb1* (red) expression in alveolar ROIs was quantified at indicated time points in mock or SARS-CoV-2 MA10-infected 1-year-old female BALB/c mice. **(J)** *Tgfb1* expression was measured by RNA-ISH in subpleural diseased regions in a SARS-CoV-2 MA10 infected mouse at 30 dpi compared to mock. Scale bars = 1 mm (low power) and 100 μm (high power). DSP Q3 normalized count graphs in (**C and I**) represent all ROIs selected with each unique color representing one animal, bars represent average value of each group with error bars representing standard error of the mean . RNA-ISH quantification graphs **(E and H)** represent average value of each group with error bars representing standard error of the mean.

In diseased alveolar ROIs at 15 and 30 dpi, multiple genes involved in adaptive immune signaling and extracellular matrix deposition were highly up-regulated, consistent with a wound repair/profibrotic environment (**Fig. 6A and B**). Recent human COVID-19 autopsy and transplant lung studies identified abundant interstitial pro-fibrotic monocyte-derived macrophages characterized by increased expression of *SPP1*, *MMP9*, and *CTSZ* (*16*, *47*, *49*). These macrophage features, coupled with up-regulated extracellular matrix remodeling (*SPARC*, *CTSK*) and macrophage-colony stimulating factor signaling genes (*CSF1*, *CSF1R*), defined a profibrotic macrophage archetype in human IPF samples (*50*). Our DSP analyses identified features associated with this profibrotic macrophage archetype in diseased alveolar ROIs at 15 and 30 dpi, including increased *Spp1*, *Sparc, and Csf1r* expression (**Fig. 6C**). RNA-ISH confirmed a persistent increase in *Spp1* expression in SARS-CoV-2 MA10-infected mice after 7 dpi (**Fig. 6D and E**). These chronic fibrotic manifestations were consistent with IHC and flow cytometry data demonstrating increased interstitial macrophage populations during chronic SARS-CoV-2 MA10 infection (**fig. S5H**). Additionally, adaptive immune cell signatures, such as immunoglobulin (*Igha*, *Igkc*, *J chain*) and major histocompatibility complex (MHC) II (*H2-Ea*, *H2-Eb1*, *H2-Ab1*) genes, were up-regulated in diseased alveolar ROIs at 30 dpi (**Fig. 6B**), consistent with the accumulation of interstitial macrophages and CD19^+^ B cells observed by immunohistochemistry and flow cytometry (**fig. S5A, G, and H**).

In parallel, we characterized genes from SARS-CoV-2 MA10-infected mice associated with human IPF (*50*). Hierarchical clustering of alveolar ROIs (**Fig. 6F** **, data file S2**) demonstrated enrichment of extracellular matrix-related genes (*Col1a1*, *Fbn1*, and *Fn1*) in mouse alveolar disease ROIs at 15 and 30 dpi (**Fig. 6A and B, F**). IHC and RNA-ISH confirmed increased expression of Col1a1 protein and *Fn1* transcripts in the subpleural pro-fibrotic alveolar regions at 15 and 30 dpi (**Fig. 4D** **,** **6G and H**). TGF-β is likely a central pro-fibrotic growth factor in IPF (*51*), and although DSP data demonstrated an up-regulated TGF-β signaling pathway (**Fig. 3C**), *Tgfb1* expression itself was not up-regulated in alveolar diseased versus intact ROIs at 15 and 30 dpi (**Fig. 6I**), suggesting post-translational rather than transcriptional regulation of TGF-β signaling (*52*). RNA-ISH revealed aggregated *Tgfb1* expression in alveolar fibrotic regions, associated with lymphocyte accumulation, in SARS-CoV-2 MA10-infected mice at 30 dpi (**Fig. 6J**). These data suggest common pathways are activated in the development of IPF in humans and our mouse model of PASC.

### Mouse SARS-CoV-2 MA10 infection recapitulates features of human lungs from fatal COVID-19 cases.

We next compared mouse and published human data to a human COVID-19 autopsy cohort. Analyses of human COVID-19 autopsy by DSP, histology scoring, and immunohistochemistry revealed biological networks and processes modified by COVID-19 disease that were recapitulated in SARS-CoV-2 MA10-infected mice (**fig. S8, data file S6**). Given the small number of patients, heterogeneity of time between disease onset and death, and patient variability, pathway analyses of COVID-19 lung samples were performed rather than longitudinal or patient-based analyses. These analyses revealed: 1) transcriptional alterations in DSP COVID-19 ROIs that separated from non-COVID-19 ROIs as indicated by PCA (**fig. S8A**); 2) histological evidence of chronic inflammation and organizing lung injury with up-regulation of networks containing type I and II interferon-stimulated as well as IL-6-driven inflammation signatures (**fig. S8B, data file S2**); 3) up-regulation of collagen and fibrotic gene signatures containing multiple human IPF genes [*COL1A1*, *COL15A1*, *FBN1*, *FN1, TNC*, consistent with mouse gene signatures; (**Fig. 6A, B, F, and H** **)**] with increased collagen and SMA protein shown by IHC (**fig. S8C and D**); 4) evidence of complement activation; an 5) evidence for altered alveolar architecture as indicated by down-regulation of AT1/endothelial networks and AT2 gene markers. Note, some findings differed from mice. For example, ciliated and *TP63/MUC5AC* networks were enriched in some COVID-19 lungs, which are consistent with histopathologic IPF features that exhibit infiltration of fibrotic alveoli with airway basal cells and “honeycombing cysts” lined by mucus producing ciliated epithelia (*51*, *53*). The absence of this finding in the mouse may reflect a dearth of basal cells in the bronchiolar region of mice or unknown preexisting lung disease in patients with COVID-19 (*31*, *53*, *54*).

### EIDD-2801 reduces chronic pulmonary lesions and nintedanib decreases peak fibrotic disease.

**Fig. 7.**
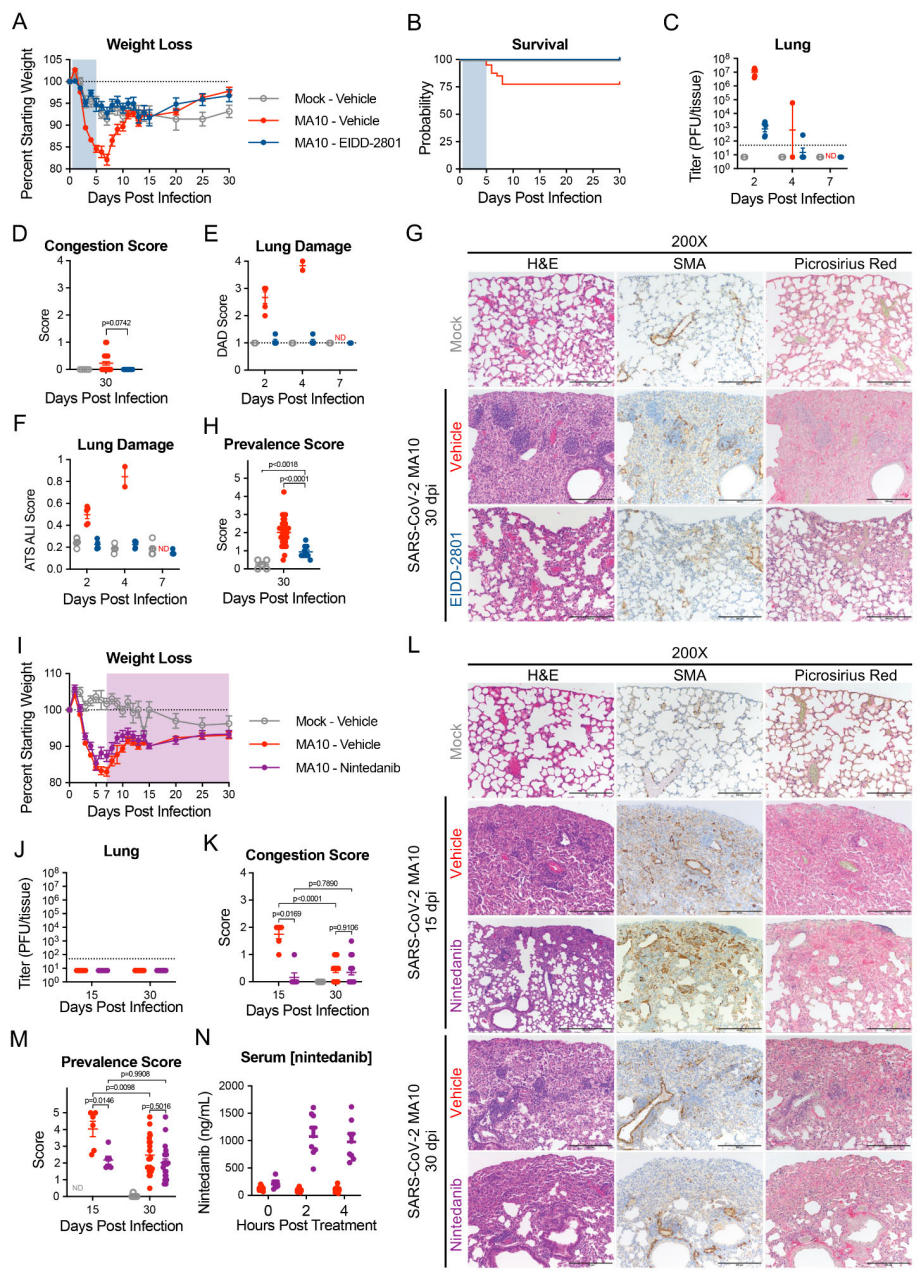
Direct acting antiviral EIDD-2801 prevents lung damage and anti-fibrotic Nintedanib reduces peak disease in SARS-CoV-2 infected aged mice. 1-year-old female BALB/c mice were infected with 10^3^ PFU of SARS-CoV-2 MA10 (n=50) or PBS (n=5) then treated with EIDD-2801 (n=10) (500 mg/kg BID) or vehicle (n=45) starting at 12 hours post infection until 5 days post infection (blue shaded region). Animals were monitored for **(A)** weight loss and **(B)** survival. **(C)** Log transformed infectious virus lung titers were assayed at selected time points. Dotted line indicates LOD and undetected samples are plotted at half the LOD. **(D)** Pathology scores of mice as measured by lung congestion at time of harvest. **(E)** lung damage measured by evaluation of H&E staining for diffuse alveolar damage, **(F)** and acute lung injury. ND, not determined. **(G)** Histopathological analysis of lungs at indicated time points is shown. H&E indicates hematoxylin and eosin. SMA indicates DAB-labeling (brown) immunohistochemistry for α-smooth muscle actin. Picrosirius Red staining (bright pink-red) highlights collagen fibers. Scale bars represents 100 μm for 200X images. **(H)** Disease incidence scoring at indicated time points: 0 = 0% of total area of examined section, 1 = less than 5%; 2 = 6 to 10%; 3 = 11 to 50%; 4 = 51 to 95%; 5 = greater than 95%. 1-year-old female BALB/c mice were infected with 10^3^ PFU of SARS-CoV-2 MA10 (n=90) or PBS (n=5) then treated with Nintedanib (n=45) or vehicle (n=50) starting at 7 days post infection until designated harvest date (shaded purple region). Animals were monitored for **(I)** weight loss and **(J)** survival. **(K)** Gross pathology scores of mice were measured by lung congestion at time of harvest. (**L**) Histopathological analysis of lungs at indicated time points were stained as in (G). Image scale bars represents 100 μm for 200X images. **(M)** Disease incidence scoring at indicated time points was recorded as in (H). **(N)** Serum nintedanib concentrations were measured. Graphs represent individuals collected at each time point in (B to F, H, J to L, and N), with the average value for each treatment and error bars representing standard error of the mean shown. Kruskal-Wallis (D, H) and two-way ANOVA (K, L) were performed and p-values are given with comparisons on each graph. Mock infected animals represented by open gray circles, vehicle treated SARS-CoV-2 MA10 infected animals are represented by closed red circles, EIDD-2801-treated SARS-CoV-2 infected animals are represented by closed blue circles, and nintedanib-treated SARS-CoV-2 infected animals are represented by closed purple circles.

EIDD-2801 (molnupiravir) is an FDA-approved direct-acting antiviral that rapidly clears SARS-CoV-2 infection in mice and humans (*55*, *56*). We treated infected 1-year-old female BALB/c mice with EIDD-2801 or vehicle twice daily from 12 hours post-infection to 5 dpi and followed survivors through 30 dpi. As reported (*55*), EIDD-2801 administration reduced weight loss, mortality, virus titers, gross lung congestion, diffuse alveolar damage (DAD) and ALI during the acute phase of infection (**Fig. 7A to F**). At 30 days, profibrotic disease prevalence was reduced compared to vehicle controls (**Fig. 7G and H**).

Nintedanib is an FDA approved anti-fibrotic therapeutic agent that prevents IPF progression in humans (*57*, *58*). Nintedanib inhibits platelet-derived growth factor (PDGF), fibroblast growth factor (FGF), and vascular endothelial growth factor (VEGF) receptors and interferes with fibroblast proliferation, migration, differentiation, and secretion of extracellular matrices (*59*). Older BALB/c mice that received nintedanib continuously from 7 dpi showed no differences in weight loss or recovery compared to vehicle treated mice through 30 dpi (**Fig. 7I**). Nintedanib treatment beginning at 7 dpi did not affect the clearance of infectious virus by 15 dpi (**Fig. 7J**). Nintedanib treatment, however, decreased gross tissue congestion scores, fibrotic prevalence scores, and collagen deposition, at 15 dpi compared to controls (**Fig. 7J to M**). Serum nintedanib concentrations were confirmed by ultra-high performance liquid chromatography time-of-flight (UHPLC-TOF) mass spectrometry to be within range previously reported in mice (*60*) (**Fig. 7N**).

## DISCUSSION

SARS-CoV-2 infection causes ALI, ARDS, and PASC. Although PASC encompasses non-respiratory sequelae, including cardiovascular and neurologic disease (*61*), pulmonary manifestations are especially common, including CAP and PF (*62*, *63*). CT scans reveal chronic COVID-19 pulmonary findings as evidenced by ground glass opacities (44%) and fibrosis (21%) after acute COVID-19 infection (*64*) and fibrotic-like changes (35%) 6 months after severe human COVID-19 pneumonia (*65*). Pathology studies of COVID-19 lungs obtained at autopsy reveal similar late findings, such as CAP and PF (*53*, *66*, *67*). Accordingly, we focused our studies of PASC in the SARS-CoV-2 MA10 mouse model of COVID-19 and specifically on the pulmonary features of PASC. Currently, our understanding of PASC and COVID-19-induced CAP and PF is poor, and countermeasures are limited due to the wide spectrum of potential disease pathophysiologies. Although better studied, these limitations are also observed in infections with other viral respiratory pathogens such as influenza (*68*–*71*). Human and animal model data comparing the chronic sequelae of a spectrum of respiratory viruses should, therefore, identify unique versus shared disease manifestations and mechanisms of disease that will contribute to not only knowledge of COVID-19 PASC, but virus infection-mediated sequelae of the lung in general (*72*–*74*).

Recently, a chronic (30 dpi) SARS-CoV-2 infection model was reported in immunosuppressed, humanized mice characterized by persistent virus replication and chronic inflammation with fibrotic markers, typical of rare infections seen in immunosuppressed humans who cannot clear virus (*75*). In contrast, we report a mouse model of long-term pulmonary sequelae of SARS-CoV-2 infection that persisted after virus clearance and was more characteristic of disease outcomes seen at the general patient population. In the SARS-CoV-2 MA10 model, surviving older mice cleared infection by 15 dpi but exhibited damaged pulmonary epithelia accompanied by secretion of a spectrum of pro-inflammatory and pro-fibrotic cytokines often up-regulated in fibrotic disease in humans, including IL-1β, TNF-α, GM-CSF, TGF-β, IL-33, and IL-17A (*76*). Like humans, surviving SARS-CoV-2-infected mice developed heterogeneous, persistent pulmonary lesions of varying severity by 30 to 120 dpi (*77*–*79*), presenting with abnormally repairing AT2 cells, interstitial macrophage and lymphoid cell accumulation, myofibroblast proliferation, and interstitial collagen deposition, particularly in subpleural regions. Micro-CT detected heterogeneous subpleural opacities and fibrosis in surviving mice, similar to human studies (*64*). Although most acute cytokine concentrations returned to normal values by 30 dpi, DSP and RNA-ISH data revealed focally prolonged up-regulation of cytokine signaling, including TGF-β, in sub-pleural fibrotic regions. Importantly, similar heterogeneous cellular and fibrotic features in subpleural regions are also evident in patients with late stage COVID-19 (*80*).

SARS-CoV-2 MA10 infection principally caused acute loss of distal airway club cell (*Scgb1a1*) and alveoli AT2 cell (*Sftpc*) marker expression, phenotypes consistent with SARS-CoV-2 cellular tropism in humans (*81*). The expression of club and AT2 cell genes were variably restored by 15 dpi, as demonstrated by DSP and RNA-ISH data. We speculate that a key variable determining the ability of the alveolar region to repair, or not, reflects the capacity of surviving or residual AT2 cells to regenerate an intact alveolar epithelium. The failure of AT2 cells to replenish themselves or AT1 cells and repair alveolar surfaces in subpleural regions may reflect the intensity of SARS-CoV-2 infection. Based on data from COVID-19 autopsy lungs, an accumulation of replication-defective and pro-inflammatory (ADI/DATP/PATS) transitional cells emerge early after SARS-CoV-2 infection and may persist, associated with continued inflammation and failure of repair (*47*, *48*). Our longitudinal mouse model data support this notion as evidenced by the observation that ADI/DATP/PATS cells were detected at 2 dpi and persisted through 30 dpi in diseased, but not morphologically intact, alveolar regions. These ADI/DATP/PATS cells were notable for up-regulation of pathways associated with senescence, Hif1α, and pro-inflammatory cytokines such as IL-1β, consistent with low cycling rates, a failure to replenish AT2 and AT1 cells, and a pro-inflammatory phenotype (*45*). However, as evidenced by the return of *Sftpc* expression by 15 dpi in intact alveolar regions, a fraction of the ADI/DATP/PATS cells likely regenerated mature *Sftpc*-expressing AT2 cells. Notably, our longitudinal studies revealed that the gene expression profiles of ADI/DATP/PATS cells are dynamic over the evolution of lung disease.

As reported in humans, CD4^+^ and CD8^+^ T cell populations increased in SARS-CoV-2-diseased areas of mouse lungs, and peripheral lymphoid aggregations were a feature of chronic disease. These features were consistent across all analyses, including immunohistochemistry, DSP, and flow cytometry data. A notable macrophage feature, identified by DSP and flow cytometry data, was expansion of the interstitial macrophage population, consistent with human data (*16*). The subpleural regions exhibited the most striking histologic evidence of immunologic cell recruitment and activation of adaptive immune, hypoxia, fibrotic, and extracellular matrix pathways in association with ADI/DATP/PATS cells.

Final clues to the etiology of the late-stage alveolar CAP/PF response emerged from comparisons to infection in bronchioles. The alveolar regions exhibited persistent CAP/PF disease, particularly in subpleural regions. This finding is consistent with proposed relationships between the maximal pulmonary mechanical stretch imposed on the subpleural region during tidal breathing and activation of stretch-induced fibrotic pathways, such as TGF-β-mediated signaling, during periods of injury (*82*, *83*). In contrast, despite similar infection, bronchioles repaired without evidence of organizing or fibrotic sequelae. Bronchioles may be protected from this adverse fate by tissue-specific ISG responses to control the duration or severity of infection. In this context, several ISGs, including *Ifitm1* and *Ifitm2*, exhibited clear differences in tissue specific expression or persistence through 30 dpi. Other possible relevant variables that may favor bronchiolar repair include: 1) more “controlled” cell death, such as apoptosis; 2) a less damaged basement membrane architecture; and 3) inability of club cells to enter an intermediate, ADI/DATP/PATS cell equivalent.

Mouse models of acute and chronic viral disease are critical also for countermeasure development. Molnupiravir is one of three FDA-approved direct-acting antivirals that clear virus, reduce morbidity, mortality, and time to recovery (*55*, *84*). Early molnupiravir treatment attenuated chronic PASC in the SARS-CoV-2 MA10 mouse model. Although speculative, early direct-acting antiviral treatment may forestall chronic lung and other organ PASC manifestations. Based on preclinical studies of anti-fibrotic agents in reducing the severity of PF responses to chemical agents, we tested the concept that early intervention with an anti-fibrotic agent may reduce the severity of PF following SARS-CoV-2 infection (*59*). Nintedanib administered from 7 dpi blunted maximal fibrotic responses to virus at 15 dpi, supporting the concept that early intervention with anti-fibrotic agents may attenuate post-SARS-CoV-2 severe disease trajectories. This suggests that early administration of direct-acting antivirals or antifibrotic drugs may help reduce human pulmonary fibrosis, and combination therapies may further increase efficacy and prognoses. Additional studies of other anti-fibrotic candidates and host immune modulators will be important in continuing to develop PASC treatments. COVID-19 in mice and humans represent key findings that may prove translatable to other future emerging coronavirus disease pathologies. Moreover, comparative models of viral induced chronic lung disease are needed to identify common and unique pathways associated with virus-induced CAP and are key for the development of new therapeutic options for treating PASC.

With respect for study limitations, the SARS-CoV-2 MA10 model was developed using the ancestral clinical SARS-CoV-2/USA-WA1 strain that was unable to utilize murine angiotensin converting enzyme 2 (ACE2) as a receptor and was serially passed to acquire increased virulence. Recently, variants of concern (VOC) human strains have been isolated subsequent to the USA/WA1 strain which contain spike receptor binding domain substitutions that permit direct infection of standard laboratory mice. However, similar to the parental strain of MA10, strain SARS-CoV-2 MA (*85*), these VOC isolates do not cause substantial acute disease in mice, and persistent post-viral phase disease has not been reported. Our mouse adapted SARS-CoV model has been used to evaluate human neutralizing antibodies, antiviral drugs, and vaccines that have progressed through phase III human trials, resulting in FDA approved products for human use (*86*–*92*). Herein, we show that acute and chronic disease phases in SARS-CoV-2 MA10-infected mice strongly recapitulate the pulmonary pathology observed in patients with COVID-19 and provides an excellent model for studies of pathogenesis and selected countermeasures. Transgenic and vectored expression of human ACE2 mouse models are also commonly used to understand SARS-CoV-2 pathogenesis (*93*–*95*), but studies investigating the long-term pulmonary effects of infection in these models have not been reported. Additionally, this study was limited to the long-term consequences of SARS-CoV-2 MA10 infection only in female mice due to housing constraints for long term studies within our BSL3 facility. It remains unclear if sex-related effects account for long-term disease progression and recovery following SARS-CoV-2 infection in mice.

The current study provides new data for modeling chronic SARS-CoV-2 and indeed other respiratory viral pathogens. By extending the studies of SARS-CoV-2 MA10 sequelae in mice out to 120 days in 1-year-old BALB/c mice, we observed that many chronic phenotypes first observed at 15 dpi were maintained for the entire 120 d observational period, extending from acute to ongoing to chronic COVID-19 defined disease classifications used in human populations (*29*). The observation that fibrotic pulmonary disease in young BALB/c and aged C57BL/6J mice peaked at 15 dpi, but waned by 30 dpi in many animals compared to aged BALB/c mice suggests that multiple time points for extended time intervals will be required for informative studies across viral pathogens. In summary, the SARS-CoV-2 MA10 mouse model provides opportunities to longitudinally study the molecular mechanisms and pathways mediating long-term COVID-19 pulmonary sequelae as relates to human PASC and to evaluate treatments. Although future studies will be needed to determine if other chronic, extrapulmonary organ sequelae develop after acute infection, the current model supports high-priority research directions that include SARS-CoV-2 infection of transgenic lineage tracing reporter mice to define longitudinally the fates of infected club and AT2 cells, ADI/DATP/PATS cell transitions, mechanisms of cell death, and epithelial cell regeneration and repopulation following infection. This study also provides the foundation for understanding the role of sex, host genetics, and immunological contributions, through knockout and Collaborative Cross studies (*96*–*98*), in defining PASC outcomes. Additional studies should be feasible in this model to investigate the non-pulmonary sequelae of PASC, including cardiovascular, neurological, and behavior manifestations in mice. With respect to countermeasures, one-year-long clinical trials are required to assess therapeutic benefit for lung fibrosis, emphasizing the utility of the SARS-CoV-2 MA10 model to rapidly test agents that may counter the pulmonary CAP/PF effects of COVID-19 (*57*, *99*). Thus, the murine SARS-CoV-2 MA10 model permits longitudinal selection and validation of therapeutic targets, accelerated timelines, and controlled experimental settings for testing of additional therapeutic agents.

## MATERIAL AND METHODS

### Study Design

The goal of these studies was to determine the prolonged pulmonary manifestations and underlying mechanisms of SARS-CoV-2-induced lung injury in mice, with intent to highlight host pathways as possible therapeutic targets to treat post-acute sequelae of SARS-CoV-2 (PASC) in patients surviving COVID-19. Animal experiments were restricted to only female mice due to long-term housing constrains within our biosafety level 3 (BSL3) facility. Animal experimental cohorts were designed sufficiently large for at least 3 to 5 mice to be in each experimental condition and timepoint, accounting for survival rate following SARS-CoV-2 MA10 infection. Animals were randomized into experimental groups with predetermined endpoints at the start of the experiment. Mice for experimental replicates were age-matched. Experimental endpoints were chosen based on previous work understanding acute SARS-CoV-2 MA10 infection and timepoints chosen for other respiratory infections in mice. For digital spatial profiling (DSP) whole transcriptome analyses, 3 representative animals per group (mock, 2 days post infection (dpi), 15 dpi, and 30 dpi) were selected based on limitations of glass slide occupancy of the GeoMx instrument. Regions of interest (ROIs) were selected by an unblinded veterinary pathologist to ensure all tissue types of interest were included in the analysis. All animals and samples were included in downstream data analysis. RNA in situ hybridization and immunohistochemistry were utilized to validate the data obtained from DSP analyses. Representative pathology images were selected from animals that represent the mean score following blinded quantification and were taken by a veterinary pathologist. Findings from mouse DSP data were validated on samples from matched and independent samples from repeated experimental cohorts. Human autopsy samples were limited by the number of samples we were able to obtain and used to corroborate findings in mice.

### Ethics and biosafety

The generation of SARS-CoV-2 MA10 was approved for use under BSL3 conditions by the University of North Carolina at Chapel Hill Institutional Review Board (UNC-CH IBC) and by a Potential Pandemic Pathogen Care and Oversight committee at the National Institute of Allergy and Infectious Diseases (NIAID). All animal work was approved by Institutional Animal Care and Use Committee (IACUC) at University of North Carolina at Chapel Hill according to guidelines outlined by the Association for the Assessment and Accreditation of Laboratory Animal Care (AAALAC) and the U.S. Department of Agriculture (USDA) under UNC IACUC protocol 21-122. All work was performed with approved standard operating procedures and safety conditions for SARS-CoV-2, including all virologic work was performed in a high containment BSL3 facility and personnel wore powered air purifying respirators (PAPRs), Tyvek suits, and were double gloved. Our institutional BSL3 facilities have been designed to conform to the safety requirements recommended by Biosafety in Microbiological and Biomedical Laboratories (BMBL), the U.S. Department of Health and Human Services, the Public Health Service, the Centers for Disease Control and Prevention (CDC), and the National Institutes of Health (NIH). Laboratory safety plans have been submitted, and the facility has been approved for use by the UNC Department of Environmental Health and Safety (EHS) and the CDC.

### Viruses and cells

Serial in vivo passaging of parental SARS-CoV-2 MA virus (*85*) in mice lead to the plaque purification of a passage 10 clonal isolate (SARS-CoV-2 MA10) (*28*). A large working stock of SARS-CoV-2 MA10 was generated by passaging the plaque purified clonal isolate sequentially on Vero E6 cells at 37°C (passage 3, SARS-CoV-2 P3). SARS-CoV-2 MA10 P3 was used for all in vivo experiments. Vero E6 cells were cultured in Dulbecco’s modified Eagle’s medium (DMEM, Gibco) with the addition of 5% Fetal Clone II serum (Hyclone) and 1X antibiotic/antimycotic (Gibco). Working stock titers were determined using plaque assay by adding serially diluted virus to Vero E6 cell monolayers. After incubation, monolayers were overlayed with media containing 0.8% agarose. After 72 hours, Neutral Red dye was used to visualize plaques.

### In vivo infection

BALB/c mice used in this study were purchased from Envigo (BALB/cAnNHsd; strain 047) and C57BL/6J mice were purchased from Jackson Labs (strain 000664). Because of BSL3 animal housing constraints magnified by the duration of these studies, only female mice were studied. For intranasal infection, mice were anesthetized using a mixture of ketamine and xylazine. 10^4^ plaque forming units (PFU) SARS-CoV-2 MA10 diluted in phosphate-buffered saline (PBS) were used for inoculation of 10-week-old BALB/c and 1-year-old C57BL/6J mice. 1-year-old BALB/c mice were infected with 10^4^ PFU SARS-CoV-2 MA10. Weight loss and morbidity were monitored daily as clinical signs of disease whereas lung function was assessed at indicated time points using whole body plethysmography (WBP; DSI Buxco respiratory solutions, DSI Inc.). Lung function data was acquired as previously described (*100*) by allowing mice to acclimate in WBP chambers for 30 min and a data acquisition time of 5 min. Data was analyzed using FinePointe software.

At indicated harvest time points, randomly assigned animals were euthanized by an overdose of isoflurane and samples for analyses of titer (caudal right lung lobe) and histopathology (left lung lobe) were collected. Animals recorded as “dead” on non-harvest days were either found dead in cage or were approaching 70% of their starting body weight, which resembles the criteria for humane euthanasia defined by respective animal protocols. Viral titers in lungs were determined by plaque assay for which caudal right lung lobes were homogenized in 1mL of PBS and glass beads, monolayers of Vero E6 cells inoculated, and 72 hours after incubation stained with Neutral Red dye for visualization of plaques as described above.

### Disease incidence scoring

Profibrotic disease incidence was scored by a blinded veterinary pathologist using serial hematoxylin and eosin (H&E) and Picrosirius Red stained slides. Ordinal scoring was defined by percent of total parenchyma affected on the sampled section: 0 = 0% of total parenchyma, 1 = less than 5%; 2 = 6 to 10%; 3 = 11 to 50%; 4 = 51 to 95%; 5 = greater than 95%. Instances of rare and isolated alveolar septa with gentle fibrotic changes were excluded from scoring.

### Chemokine and Cytokine analysis

Chemokine and cytokine profiles of serum and lung samples were assessed using Immune Monitoring 48-plex mouse ProcartaPlex Panel kits (Invitrogen). Briefly, 50 μL of either a 1:4 dilution of serum or 50 μL straight clarified lung homogenate were incubated with magnetic capture beads containing analyte specific antibodies. After washing, 96-well plates containing samples and magnetic beads were incubated with detection antibodies and streptavidin conjugated to phycoerythrin (SA-PE). Results were collected using a MAGPIX machine (Luminex) and quantification was achieved by comparing to a standard curve; both were done in xPONENT software. Values below limit of detection (LOD) were set to LOD and hierarchical clustering heatmaps were generated with the Bioconductor R package, *ComplexHeatmap*, after scaling the values across samples.

### Preparation of lung cell suspensions for flow cytometric analysis

Enzymatic digestion of lung tissue was performed by intratracheal instillation using a 20-gauge catheter of 1 mL of 5 mg/mL collagenase I (Worthington Biochemical Corp) and 0.25 mg/mL DNase I (Sigma-Aldrich) prepared in RPMI-1640 media (Life Technologies) prior to instilling 0.5 mL of 1% (wt/vol) low melting agarose (Amresco), similar to previous protocols (*101*). Lung were then incubated at 37°C for 30 min. Lung were then minced and triturated through a 5 mL syringe. Cell suspensions were then filtered through a 50 mL conical 100 μM filter (Thermo Fisher Scientific) before red blood cell lysis and stained as previously described (*101*).

### Multi-color flow cytometry

The prepared lung cells were suspended in approximately 1 mL of PBS buffer supplemented with 1.5% (w/v) bovine serum albumin (Sigma-Aldrich) and 2 mM EDTA (Sigma-Aldrich). The total cell count determined by hemocytometer with trypan blue (VWR). For each sample, 1.5 × 10^6^ cells first underwent Fc receptor blockade with rat anti-mouse FcγRIII/II receptor (CD16/32; BD Biosciences). After Fc receptor blocking for 5 min on ice, cells were surface stained using antibodies listed in **table S1** and as previously described (*101*). For intracellular staining, the cells underwent fixation and permeabilization with the Foxp3/Transcription Factor Staining Buffer Set (eBioscience). Fixed and permeabilized single cells suspensions were subsequently stained with intracellular antibodies to characterize differences in specific populations. Neutrophils and macrophage subpopulations were identified through gating, as demonstrated in prior reports (*101*, *102*) and adapted from previously published methods (*103*).

Flow cytometry was performed using a Cytoflex flow cytometer (Beckman Coulter) and analyzed using CytExpert (Beckman Coulter) software. To determine the total number of a specific population in the lung, we first calculated the population’s percentage with respect to the total live single cell population. Next, we multiplied this percentage to the total cell count as determined by hemocytometer measurements to calculate the specific population’s total number per mouse lung.

### Specimen CT imaging

Phosphotungstic acid (PTA) staining was performed to increase soft tissue conspicuity for specimen computed tomography (CT) imaging. Lungs were inflated and fixed with 10% formalin at 20 cm H_2_O pressure for seven days. Samples were initially washed 3X in 70% EtOH in 50 mL non-reactive tubes prior to staining. Each lung was then immersed in 0.3% (w/v) Phosphotungstic acid hydrate (Sigma-Aldrich P4006) in 70% EtOH for seven days on an oscillating table. They were subsequently air dried prior to imaging.

Specimen CT scanning of the dried lungs was performed on a Sanco μCT 40 (ScanCo Medical AG). Imaging was performed at 70kVP at 114 μA current and 200 ms integration time. Images were reconstructed using a conebeam algorithm at 16 μm voxel size in a DICOM file format. Images were viewed with ImageJ and analyzed by blinded radiologists (M.K.S. and Y.Z.L).

### RNA-ISH, IHC, and quantification

For histopathological analyses on mouse lung tissue sections, left lung lobes were stored in 10% phosphate buffered formalin for at least 7 days before transferring out of the BSL3 facility for further processing. Histopathological scoring was performed after tissue samples were embedded with paraffin, sectioned, and stained. IHC was performed on paraffin-embedded lung tissues that were sectioned at 5 μm. This IHC was carried out using the Leica Bond III Autostainer system. Slides were dewaxed in Bond Dewax solution (AR9222) and hydrated in Bond Wash solution (AR9590). Heat induced antigen retrieval was performed for 20 min at 100°C in Bond-Epitope Retrieval solution 2, pH-9.0 (AR9640). After pretreatment, slides were incubated with primary antibodies (**table S1**) for 1 hour followed with Novolink Polymer (RE7260-K) secondary. Antibody detection with 3,3′-diaminobenzidine (DAB) was performed using the Bond Intense R detection system (DS9263). Stained slides were dehydrated and coverslipped with Cytoseal 60 (8310-4, Thermo Fisher Scientific).

RNA-ISH was performed on paraffin-embedded 5 μm tissue sections using the RNAscope Multiplex Fluorescent Assay v2 or RNAscope 2.5 HD Reagent Kit according to the manufacturer’s instructions (Advanced Cell Diagnostics). Briefly, tissue sections were deparaffinized with xylene and 100% ethanol twice for 5 min and 1 min, respectively, incubated with hydrogen peroxide for 10 min and in boiling Target Retrieval Reagent (Advanced Cell Diagnostics) for 15 min, and then incubated with Protease Plus (Advanced Cell Diagnostics) for 15 min at 40°C. Slides were hybridized with custom probes at 40°C for 2 hours, and signals were amplified according to the manufacturer’s instructions.

Stained mouse tissue sections were scanned and digitized by using an Olympus VS200 slide scanner. Images were imported into Visiopharm Software (version 2020.09.0.8195) for quantification. Lung tissue and probe signals for targeted genes detected by RNA-ISH were quantified using a customized analysis protocol package to 1) detect lung tissue using a decision forest classifier, 2) detect the probe signal based on the intensity of the signal in the channel corresponding to the relevant probe. The same methodology was applied to quantify CD4^+^ and CD8^+^ cells identified by IHC. Positive signals for CD4^+^ cells were determined using contrast of red-blue channels at a determined threshold to exclude background, similarly, CD8^+^ cells were determined using contrast of green-blue channels. All slides were analyzed under the same conditions. Results were expressed as the area of the probe relative to total lung tissue area.

Paraffin-embedded mouse and human tissue sections (5 μm) were used for fluorescent IHC staining. According to the previously described protocol (*104*) sections were baked at 60°C for 2 to 4 hours followed by a deparaffinization step including xylene and graded ethanol. Antigen retrieval was achieved after rehydration by boiling slides in 0.1M sodium citrate at pH 6.0 in a microwave. Slides were allowed to cool down and rinsed with distilled water before quenching of endogenous peroxidase was performed with 0.5% hydrogen peroxide in methanol for 15 min. After a PBS wash, slides were blocked with 4% normal donkey serum for 60 min at room temperature followed by incubation with primary antibodies (**table S1**), where antibodies were diluted in 4% normal donkey serum in PBS with 0.1% Tween 20 (PBST), at 4°C overnight. Isotype control (species-matched gamma globulin) was diluted in the same manner as the primary antibody. Slides were incubated for 60 min at room temperature with secondary antibodies after being washed in PBST. Reduction of background staining was achieved by utilization of Vector TrueVIEW Autofluorescence Quenching Kit (Vector laboratories). Tissue sections were covered in glass coverslips by adding ProLong Gold Antifade Reagent with 4’,6-diamidino-2-phenylindole (DAPI, Invitrogen). Stained human tissue sections were scanned and digitized by using an Olympus VS200 slide scanner.

### GeoMx digital spatial profiling

Five μm-thick FFPE sections were prepared using the RNAscope and DSP combined slide prep protocol from NanoString Technologies. Prior to imaging, mouse tissue morphology was visualized by IHC for CD45 and RNAscope for SARS-CoV-2 RNA, and DNA was visualized with 500 nM Syto83. Human tissue morphology was visualized by IHC for the immune cell marker CD45 and epithelial cell marker panCK/Syto83 and for KRT5 (IHC) and SARS-CoV-2 (RNA) on serial sections. Mouse or Human Whole Transcriptome Atlas probes targeting over 19,000 targets were hybridized, and slides were washed twice in fresh 2X saline-sodium citrate (SSC) then loaded on the GeoMx Digital Spatial Profiler (DSP). In brief, entire slides were imaged at 20X magnification and 6 to 10 regions of interest (ROI) were selected per sample. ROIs were chosen based on serial H&E-stained sections and morphology markers (mouse: DNA/CD45 IHC/SARS-CoV-2 RNA; human: CD45/PanCK/Syto83 IHC and SARS-CoV-2 RNA/KRT5/DAPI IHC) on serial sections by an unblinded veterinary pathologist (S.A.M.). The GeoMx then exposed ROIs to 385 nm light (UV) releasing the indexing oligos and collecting them with a microcapillary. Indexing oligos were then deposited in a 96-well plate for subsequent processing. The indexing oligos were dried down overnight and resuspended in 10 μL of diethyl pyrocarbonate (DEPC)-treated water.

Sequencing libraries were generated by PCR from the photo-released indexing oligos and ROI-specific Illumina adapter sequences and unique i5 and i7 sample indices were added. Each polymerase chain reaction (PCR) used 4 μL of indexing oligos, 4 μL of indexing primer mix, and 2 μL of NanoString 5X PCR Master Mix. Thermocycling conditions were 37°C for 30 min, 50°C for 10 min, 95°C for 3 min; 18 cycles of 95°C for 15 s, 65°C for 1 min, 68°C for 30 s; and 68°C for 5 min. PCR reactions were pooled and purified twice using AMPure XP beads (Beckman Coulter, A63881) according to manufacturer’s protocol. Pooled libraries were sequenced at 2×27 base pairs and with the dual-indexing workflow on an Illumina NovaSeq.

### Analysis of mouse GeoMx digital spatial profiling data

For mouse samples, raw count, 3rd quartile (Q3) normalized count data of target genes from ROIs were provided by the vendor, which were used as input to downstream analyses (**data file S3**). Mouse Q3 normalized data were used for PCA using the R package *ade4* and visualized using *factoextra* package. Raw count data were used for differential expression analysis using the Bioconductor R package, *variancePartition* (*105*), with transformation of raw counts by voom method (*106*). The dream function from *variancePartition* allows fitting of mixed-effect models to account for ROIs obtained from the same animal, and assay slides as random-effect factors. Differentially expressed genes (DEGs) were defined as genes that passed the filters of Benjamini-Hochberg adjusted p-value < 0.05, and absolute log2 fold-change > 1. Pre-ranked gene set enrichment analysis (GSEA) was performed using the Bioconductor R package, *fgsea* (*107*), with gene set collections obtained from Gene Ontology Biological Process (*108*), and Reactome pathways (*109*). Various gene lists of interests were curated manually from published literature, and human gene symbols from references were converted into homologous mouse genes using bioDBnet (https://biodbnet-abcc.ncifcrf.gov/). Plots and hierarchical clustering heatmaps were generated using the R package, *ggplot2* (*110*), and *ComplexHeatmap* (*111*).

For the human samples, whole transcriptome analysis (WTA) and COVID-19 spike-in gene targets were assayed. FASTQ data were first converted to digital counts conversion (DCC) format. Probe outlier tests were performed on each set of negative probes (one set of negative probes for the WTA panel and one set for the COVID-19 spike-in panel). Specifically, for a given negative probe pool, the geometric mean of all counts (across all probes and all samples) was computed. A probe was identified as a low count outlier if its probe-specific geometric mean divided by the grand geometric mean was less than the threshold of 0.1. From the remaining probes, the Rosner Test was used to detect local outliers on a sample-specific case using the R package EnvStats (*112*) with parameters *k* equal to 20% of the number of negative probes and *alpha* equal to 0.01. A negative probe was considered a global outlier if it was found to be a local outlier in more than 20% of samples and was discarded from downstream analysis. For each panel pool, the negative probe geometric mean and geometric standard deviation were computed. The sample-specific limit of quantification (LOQ) was estimated from these moments by multiplying the geometric mean by the geometric SD and then squaring that quantity. Gene targets in the COVID-19 spike-in, which contain multiple probes per target, were collapsed to a single floating point value using the geometric mean. Following outlier filtering, the sequencing saturation for each sample was computed as the one minus the number of deduplicated reads divided by the number of aligned reads. One sample yielded a sequencing saturation below the 0.67 cutoff (range of other samples: 85.9 to 96.8) and was removed. Additionally, one sample had an LOQ more than 2.7 standard deviations from the mean in the WTA panel and 4.2 standard deviations from the mean in the COVID-19 spike-in pool and was removed from the analysis. Filtering gene targets was also performed. If a gene target was below LOQ in more than 10% of samples, it was filtered out. Following the above probe, sample, and target filtering steps, the data matrix was normalized using the Q3 method (see above).

Preliminary analysis of the log2 transformed and scaled Q3 normalized data identified a putative batch effect between two runs as identified using the PCA in the R package FactoMineR. The following batch correction algorithm was used before downstream data analysis. We first ensured that the batching factor was not itself confounded with Group (Healthy or COVID-19) or Region (alveolar, bronchiolar, disorganized). This was done by creating a design matrix and checking for any linearly dependent terms using the core R package *stats* (*113*). No factors were correlated with Batch using a correlation threshold of 0.3. Batch correction was performed for each gene target by modeling its log2 Q3 expression (dependent variable) in a mixed effect model that included a random intercept for the fixed portion and Batch as a random effect with random intercept. Modeling was done in the R package *lme4*. For each model, the residuals of the model were extracted and converted back to the linear scale. These residuals were then multiplied by the model’s estimated intercept (also linear scale) to shift the values to an intensity similar to the original Q3 data. To evaluate how well the above approach removed the batch effect, we regressed the first 5 principal component (PC) scores against Batch for both the Q3 as well as the batch corrected (BC) data using a series of analyses of variance (ANOVAs). Of the five PC axes, only the first was associated with the batching factor (P < 4x10^−36^; all others, P > 0.23) in the Q3 data. Following correction, no axes were associated with Batch (all P > 0.80).

### Analysis of human GeoMx digital spatial profiling data

For human samples, raw count and Q3 + batch corrected count data of target genes from ROIs were provided by the vendor (**table S2**, **data file S6**). Prior to downstream analysis, Q3 + batch corrected data were log_2_ normalized. PCAs were performed on the top 1,000 highly variable genes on the log normalize data. Coexpression network analysis was performed on 11,556 expressed genes using Weighted Gene Coexpression Network Analysis (WGCNA) R package (*114*). Differential gene and network expression between groups were evaluated under a linear mixed model approach accounting for multiple ROIs per donor using R package *Ime4*. Statistical significance of the estimates were evaluated with R package *lmerTest* (*115*), using the Satterthwaite’s degrees of freedom method. Sets of DEGs were tested for overrepresentation of the genes in the databases (GO: Biological Process, GO: Molecular Function, GO: Cellular Components, KEGG, and Reactome) using R package *enrichR (* *116* *)*. For each network, genes were selected based on the degree of correlation with the network eigengene. To cluster ROIs obtained from healthy and COVID-19 donors, hierarchical clustering was performed based on the 50 most correlated network genes from each of the 7 identified networks using ward.D2 agglomeration method. As a result, healthy ROIs were separated from COVID-19 ROIs and COVID-19 ROIs were segregated into three subtypes, including COVID 1, COVID 2, and COVID 3. Various plots and heatmaps were generated using the R packages ggplot2 (*110*) and heatmap3 (*117*).

### Histological scoring of human COVID-19 lung tissue

The H&E stained ROIs were scored by a blinded pulmonary pathologist (S.D.G.) grading each section on a semiquantitative scale between zero and three, with zero representing a normal human lung section and three representing the most severe histologic change encountered in clinical practice. The features scored in each ROI are: interstitial inflammation, airspace fibrin exudates (acute phase of lung injury), the fibroblastic/organizing-phase of lung injury and mature fibrosis. Human donor information can be found in **table S3.**

### Human lung tissue and quantification of Picrosirius Red and smooth muscle actin signals

Control lungs were obtained from lung transplant donors without any history of pulmonary disease whose lungs were unsuitable for transplant due to size mismatch provided by the University of North Carolina (UNC) Tissue Procurement and Cell Culture Core (institutional review board (IRB)-approved protocol #03-1396). COVID-19 autopsy lung tissue sections were obtained from Drs. Ross. E. Zumwalt (University of New Mexico), Edana Stroberg (Office of the Chief Medical Examiner, Oklahoma City), Alain Borczuk (Weill Cornell Medicine), and Leigh B. Thorne (UNC). Human donor information can be found in **table S3**. Early- and late-phase specimens were defined as autopsy tissues obtained ≤ 20 and > 20 days post an onset of symptoms, respectively.

Stained areas of Picrosirius Red and SMA detected by IHC in the alveolar regions were quantitated using Fiji software. Alveolar regions were randomly selected and cropped from the field. Optimized threshold value was determined by adjusting the threshold accurately representing the original images. The optimized threshold values were applied to identify Picrosirius Red or SMA signals. The Picrosirius Red or SMA-stained areas were measured and normalized to alveolar areas.

### In vivo Drug Treatment

EIDD-2801 (Emory Institute of Drug Design) was dissolved in a solution of 2.5% cremaphor (Sigma-Aldrich), 10% PEG 400 (Fisher Chemical), and 87.5% Molecular biology grade water (HyClone) by bath sonication at 37°C for 10 min, as described previously (*55*). Drug solution was made at a concentration of 62.5 mg/mL fresh daily for a final dose of 250 mg/kg per mouse (500 mg/kg BID). Mice were dosed by oral gavage with 100 μL of vehicle (2.5% cremaphor (Sigma-Aldrich), 10% PEG 400 (Fisher Chemical), and 87.5% Molecular biology grade water (HyClone)) or EIDD-2801 solution twice daily beginning at 12 hours post infection and were dosed every 12 hours until 120 hours post infection.

Nintedanib (MedChemExpress) suspension was made in Molecular Biology Grade Water (HyClone) with 1% Tween-80 (Sigma-Aldrich) fresh daily at a concentration of 15 mg/mL for a final dose of 60 mg/kg per mouse (*118*, *119*). Mice were dosed once daily by oral gavage with either 100 μL of nintedanib suspension or vehicle (Molecular Biology Grade Water (HyClone) with 1% Tween-80 (Sigma-Aldrich)) starting at 7 days post infection until final harvest at either 15 or 30 days post infection. Mouse serum was harvested at indicated time points after nintedanib administration, inactivated for BSL3 removal with 0.05% Triton-X100 and heating at 56°C, and was analyzed using ultra high-performance liquid chromatography time-of-flight mass spectrometry (UHPC-TOF MS). Samples were prepared by precipitating protein with acetonitrile (Sigma-Aldrich) containing diazepam (Cerilliant) as an internal standard. The supernatant was separated using a Flexar FX-20 UHPLC system (Perkin Elmer) with a Kinetex C18 biphenyl column (2.6 um 50 × 3 mm Phenomenex) at 45°C with 98% MS-grade water (Sigma-Aldrich), 10 mM ammonium acetate (Hagn Scientific), and 98% methanol (Sigma-Aldrich) 0.1% formic acid (Hagn Scientific) gradient elution at a flow rate of 0.6 mL/minute. The Perkin Elmer Axion2 TOF mass spectrometer operated in positive-ion electrospray ionization (ESI+) mode was used to detect accurate mass spectra of nintedanib at 540.2605 [M+H]+. The method was linear from 1 to 500 ng/mL with a lower limit of detection of 1 ng/mL. The results for nintedanib concentration in mouse serum for this study was in agreement with the serum concentrations reported previously (*60*).

### Statistical analysis

Raw data are available in Data File S7. Wilcoxon rank-sum test was used to test the difference in CD4^+^ or CD8^+^ T cells **(fig. S5C and D)**, as well as Picrosirius red- or SMA-stained areas **(fig. S8C and D)**, identified by IHC between two groups. Flow cytometry data were analyzed by Wilcoxon rank-sum test (**fig. S5E**) or ANOVA followed by Sidak’s multiple comparisons test **(fig. S5F to H)**. The difference in DSP Q3 normalized counts for targeted genes in ROIs between each condition and time point was statistically tested using a linear mixed-effect model using the R package *Ime4* (*120*), with condition and time point as fixed effects and replicate mice as random-effect factors (**Fig. 4C** **, fig. S6D and E**). Statistical significance was evaluated with the R lmerTest package (*115*), using the Satterthwarte’s degrees of freedom method. Multiple post-hoc comparisons of subgroups were performed using the R multcomp package (Hothorn T, 2008). P < 0.05 was considered statistically significant.

## Supplementary Materials

### Other Supplementary Material for this manuscript includes the following:

MDAR Reproducibility Checklist
