# Supplementary material for: SARS-CoV-2 infection produces chronic pulmonary epithelial and immune cell dysfunction with fibrosis in mice
Source: Sci Transl Med. 2022 Jul 7:eabo5070. doi: 10.1126/scitranslmed.abo5070 (PMC9273046; doi:10.1126/scitranslmed.abo5070)
Supplement: Supplementary file 1 — Figs. S1 to S8 Tables S1 to S3 [file scitranslmed.abo5070_sm.pdf]

Science Translational Medicine
AAAS

Supplementary Materials for

SARS-CoV-2 infection produces chronic pulmonary epithelial and immune cell dysfunction with fibrosis in mice

Kenneth H. Dinnon III *et al.*

Corresponding authors: Wanda K. O’Neal, wanda_o'neal@med.unc.edu; Stephanie A. Montgomery, stephanie@med.unc.edu; Richard C. Boucher, richard_boucher@med.unc.edu; Ralph S. Baric, rbaric@email.unc.edu

DOI: 10.1126/scitranslmed.abo5070

The PDF file includes:

Figs. S1 to S8
Tables S1 to S3

Other Supplementary Material for this manuscript includes the following:

MDAR Reproducibility Checklist
Data files S1 to S7

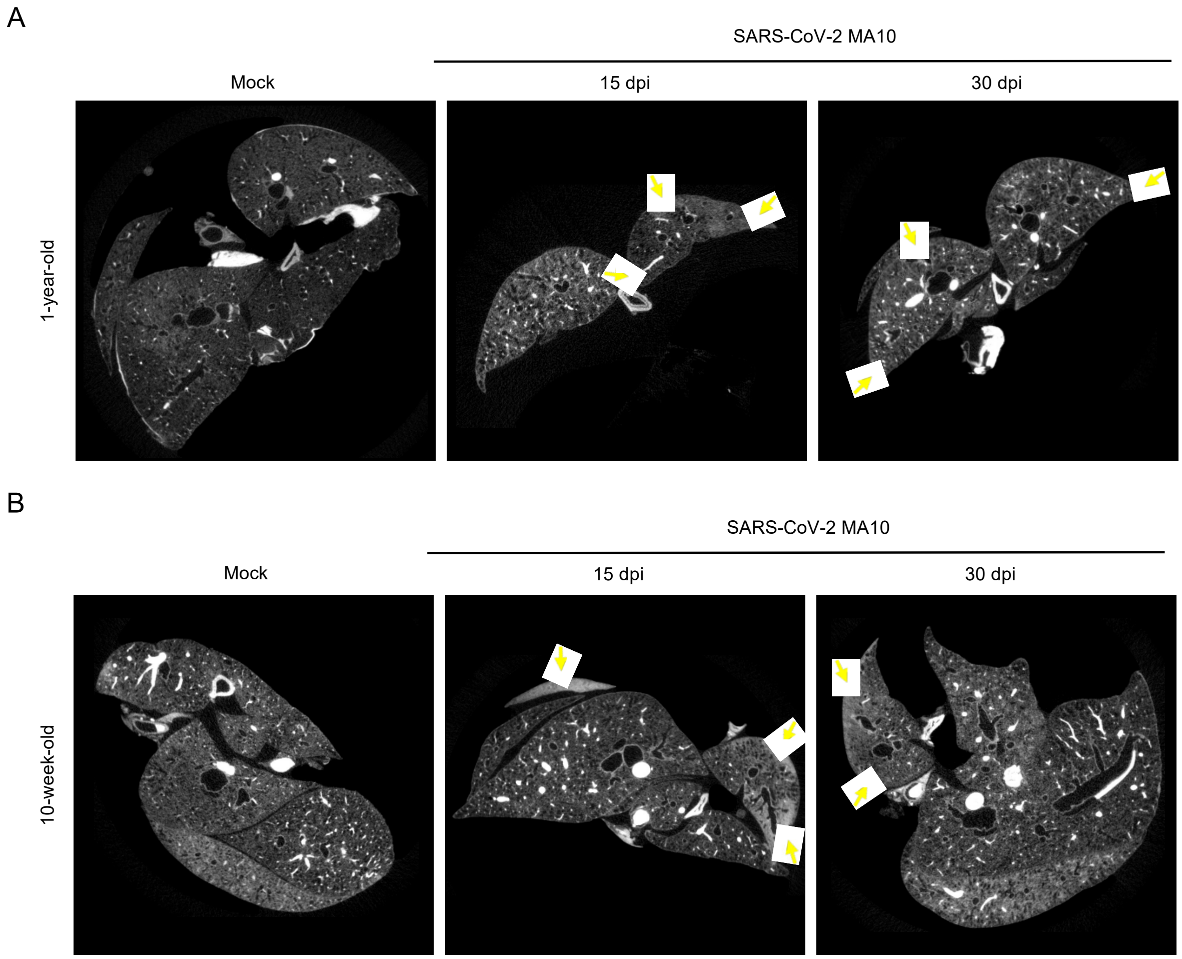

Fig. S1. Micro-computed tomography (CT) scans of mouse lungs reveal pulmonary disease. Representative images of specimen micro-CT scans are shown for **(A)** 1-year-old female mice and **(B)** 10-week-old female mice that were either mock-treated or infected with 10^3 or 10^4 PFU of SARS-CoV-2 MA10, respectively, and imaged at 15 and 30 days post infection (dpi). Arrows indicate areas of dense ground glass opacity and consolidation representing peripheral fibrosis.

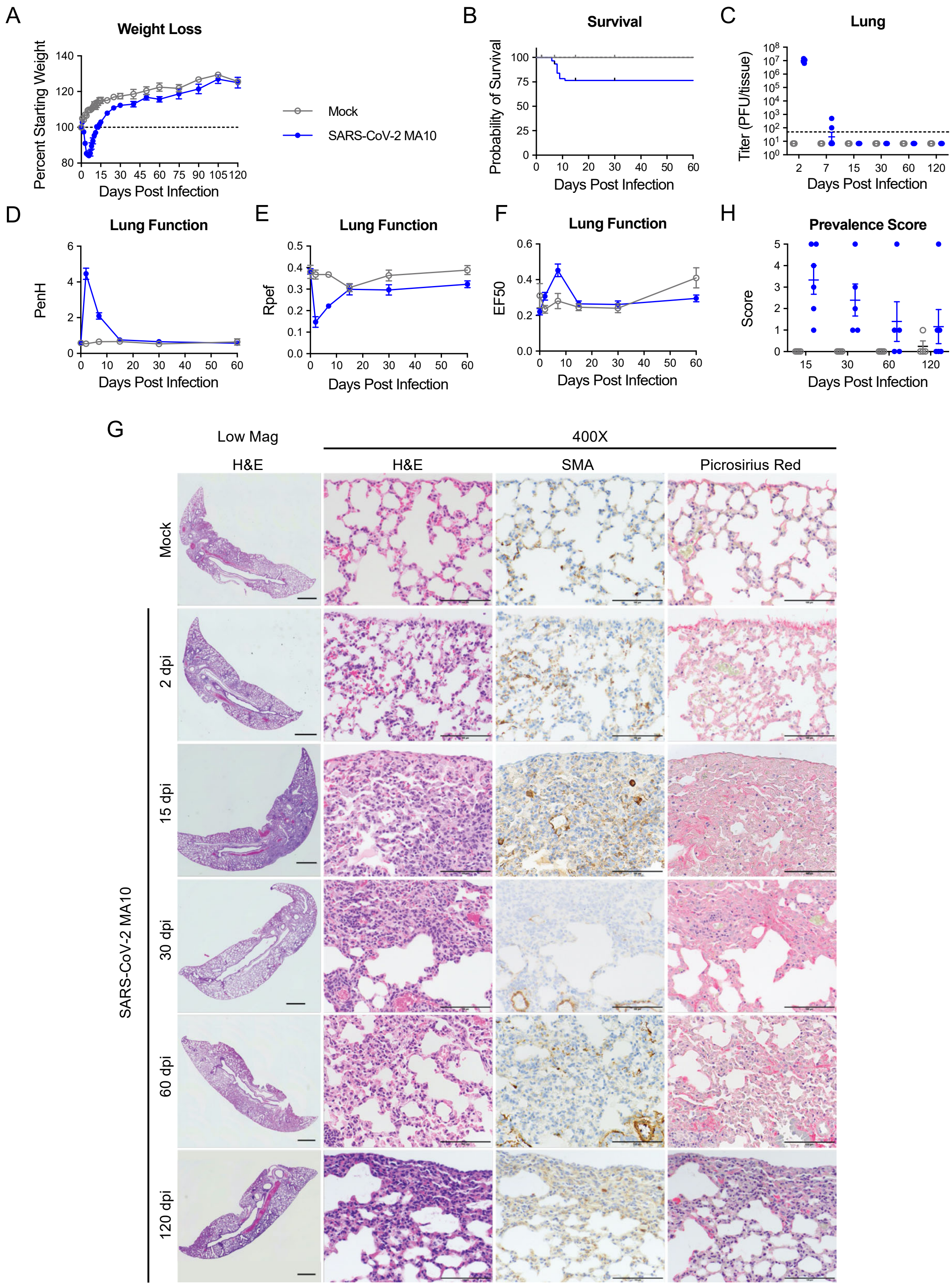
A
Weight Loss
Percent Starting Weight
Days Post Infection
Mock
SARS-CoV-2 MA10
B
Survival
Probability of Survival
Days Post Infection
C
Lung
Titer (PFU/tissue)
Days Post Infection
D
Lung Function
PenH
Days Post Infection
E
Lung Function
Rpef
Days Post Infection
F
Lung Function
EF50
Days Post Infection
H
Prevalence Score
Score
Days Post Infection
G
Low Mag
400X
H&E
H&E
SMA
Picrosirius Red
Mock
SARS-CoV-2 MA10
2 dpi
15 dpi
30 dpi
60 dpi
120 dpi

7

Fig. S2. SARS-CoV-2 MA10 infection causes lung damage in young surviving mice. 10-week-old female BALB/c mice were infected with 10^4 plaque-forming units (PFU) SARS-CoV-2 (n=66) MA10 or phosphate-buffered saline (PBS, n=24) and monitored for **(A)** percent starting weight and **(B)** survival. **(C)** Log transformed infectious virus lung titers were assayed at indicated time points. The dotted line represents limit of detection. Undetected samples are plotted at half the limit of detection. PFU, plaque-forming units. **(D to F)** Lung function was assessed by whole body plethysmography for **(D)** enhanced pause (PenH), **(E)** ratio of time to peak expiratory flow relative to expiratory time (Rpef), and **(F)** mid-tidal expiratory flow (EF50). **(G)** Histopathological analysis of lungs are shown at indicated time points. H&E: hematoxylin and eosin. SMA: 3,3'-diaminobenzidine- (DAB) labeling (brown) immunohistochemistry for α-smooth muscle actin. Picrosirius Red staining (bright pink-red) highlights collagen fibers. Image scale bars represents 1000 μm for low magnification and 100 μm for 400X images. **(H)** Disease incidence scoring is shown for the indicated time points: 0 = 0% of total parenchyma; 1 = less than 5%; 2 = 6 to 10%; 3 = 11 to 50%; 4 = 51 to 95%; 5 = greater than 95%. In **(C and H),** graphs represent individuals necropsied at each timepoint. Data in **(A to H)** are shown as mean ± standard error of the mean. Mock infected animals represented by open gray circles and SARS-CoV-2 MA10 infected animals are represented by closed blue circles.

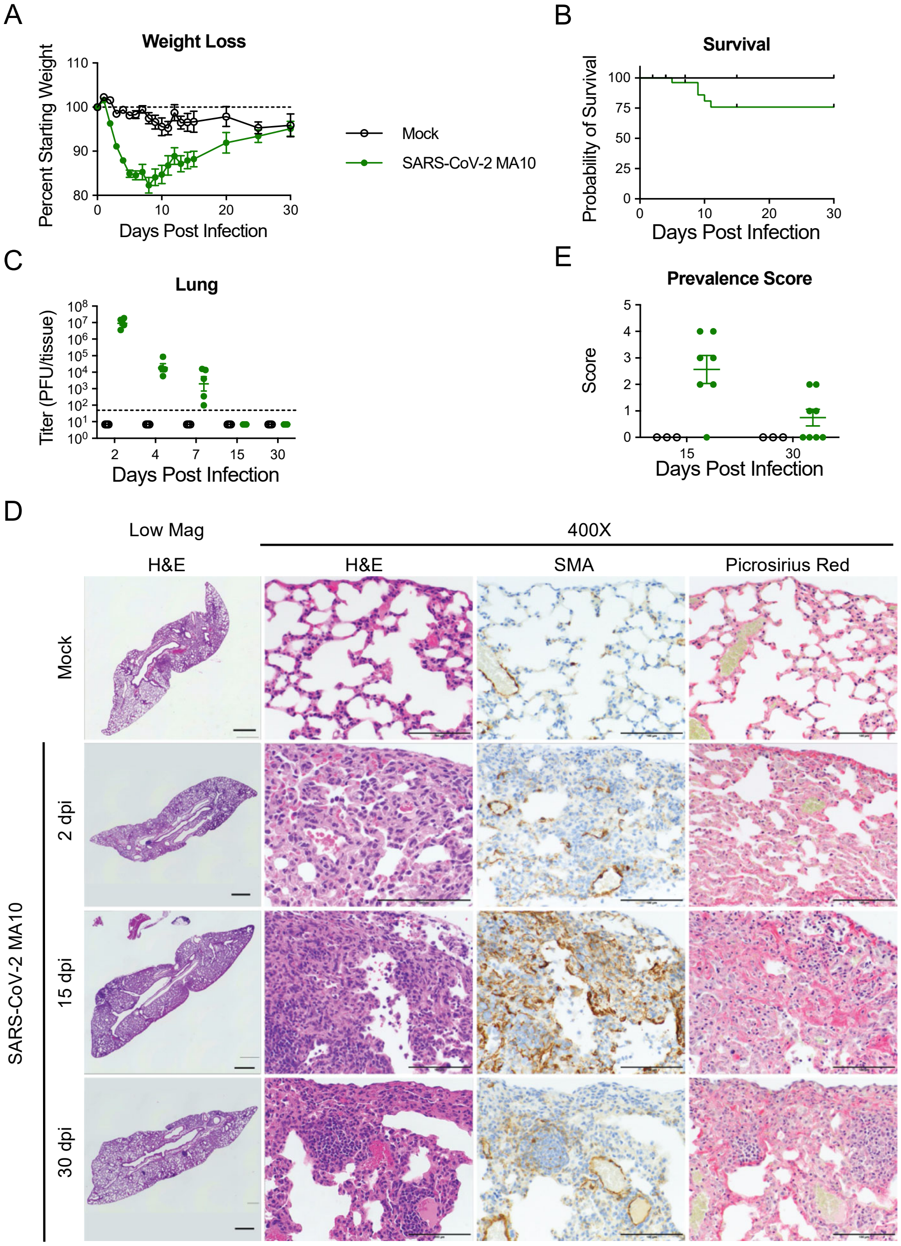

Fig. S3. SARS-CoV-2 infection causes lung damage in surviving aged C57BL/6J mice. 1-year-old female C57BL/6J mice were infected with 10^4 PFU SARS-CoV-2 MA10 (n=35) or PBS (n=15) and monitored for **(A)** percent starting weight and **(B)** survival. **(C)** Log transformed infectious virus lung titers were assayed at indicated time points. Dotted line represents limit of detection. Undetected samples are plotted at half the limit of detection. **(D)** Histopathological analysis of lungs at indicated time points. H&E:

hematoxylin and eosin. SMA: immunohistochemistry for α-smooth muscle actin. Picrosirius Red staining highlights collagen fibers. Image scale bars represents 1000 μm for low magnification and 100 μm for 400X images. **(E)** Disease incidence scoring at indicated time points: 0 = 0% of total area of examined section; 1 = less than 5%; 2 = 6 to 10%; 3 = 11 to 50%; 4 = 51 to 95%; 5 = greater than 95%. Graphs represent individuals necropsied at each timepoint **(C and D)**, with the average value for each treatment and error bars representing standard error of the mean **(A to D)**. Mock infected animals are represented by closed black circles and SARS-CoV-2 MA10 infected animals are represented by closed green circles.

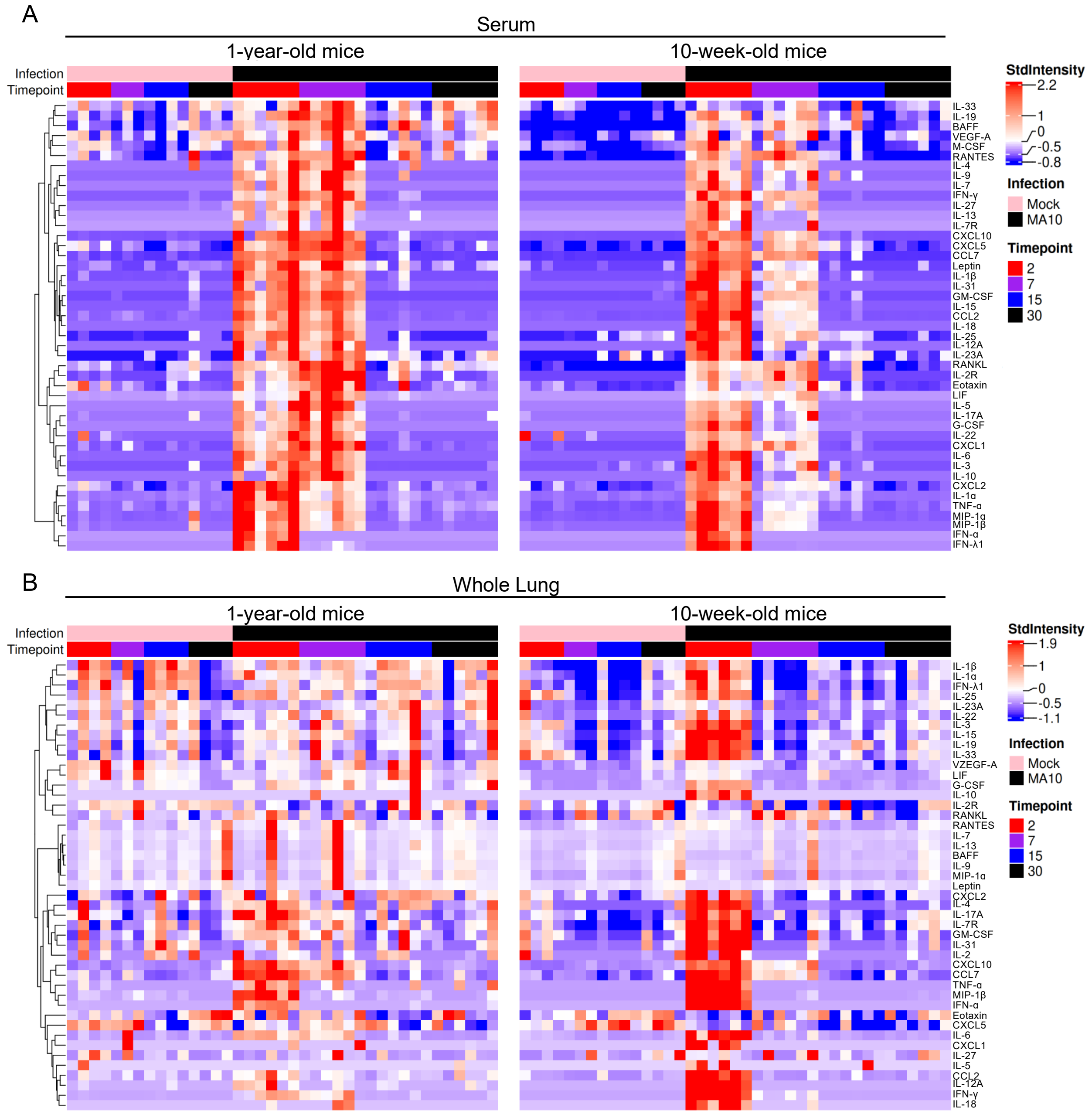

Fig. S4: SARS-CoV-2 MA10 induces local and systemic cytokine and chemokine responses. Heatmaps of cytokine and chemokine protein concentrations in lung tissue homogenate **(A)** or serum **(B)** in 1-year-old or 10-week-old female mock or SARS-CoV-2 MA10 infected mice at 2, 7, 15, and 30 dpi.

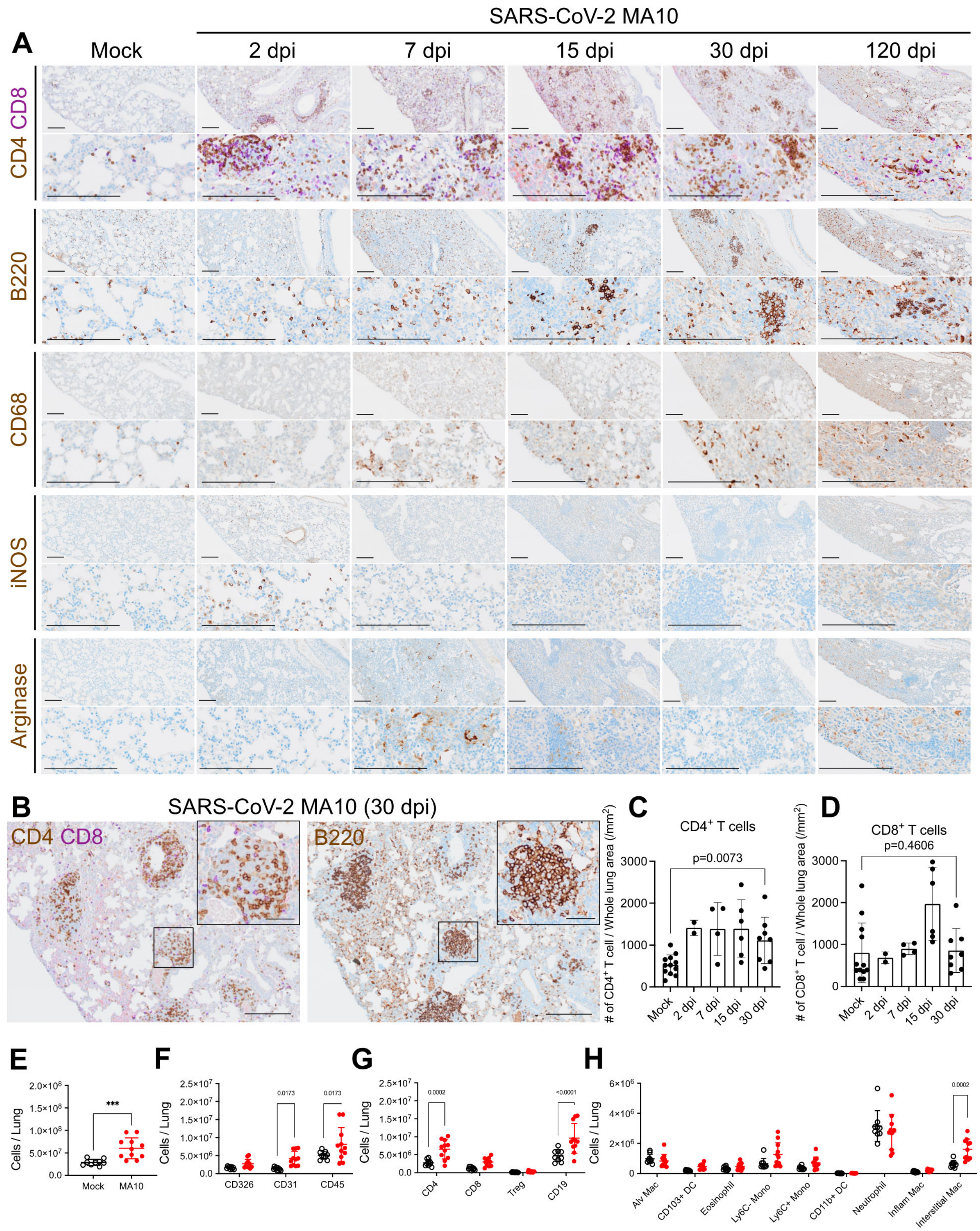

SARS-CoV-2 MA10
A
Mock
2 dpi
7 dpi
15 dpi
30 dpi
120 dpi
CD4 CD8
B220
CD68
iNOS
Arginase
B
SARS-CoV-2 MA10 (30 dpi)
CD4 CD8
B220
C
CD4+ T cells
p=0.0073
# of CD4+ T cell / Whole lung area (/mm2)
D
CD8+ T cells
p=0.4606
# of CD8+ T cell / Whole lung area (/mm2)
Mock
2 dpi
7 dpi
15 dpi
30 dpi
E
Cells / Lung
***
Mock
MA10
F
CD326
CD31
CD45
0.0173
0.0173
G
CD4
CD8
Treg
CD19
0.0002
<0.0001
H
Alv Mac
CD103+ DC
Eosinophil
Ly6C- Mono
Ly6C+ Mono
CD11b+ DC
Neutrophil
Inflam Mac
Interstitial Mac
0.0002

43

Fig. S5: SARS-CoV-2 MA10 induces robust immune cell infiltration. (A) Immunohistochemistry of CD4$^+$ (brown) and CD8$^+$ T (purple) cells, B220$^+$ B cells, CD68$^+$ macrophages, and inducible nitric oxide synthase (iNOS)$^+$ M1 and Arginase$^+$ M2 macrophages is shown for lungs from 1-year-old female BALB/c mice at indicated time points after SARS-CoV-2 MA10 infection compared to mock. Scale Bars = 200 µm. **(B)** Immunohistochemistry is shown depicting alveolar lymphocyte aggregates consisting of CD4$^+$, CD8$^+$, and B220$^+$ B cells at 30 dpi. Scale Bars = 200 µm (low power) and 50 µm (insets). **(C and D)** Quantification of **(C)** CD4$^+$ and **(D)** CD8$^+$ T cells is shown based on immunohistochemistry **(A)**. Number of **(C)** CD4$^+$ and **(D)** CD8$^+$ cells were counted and normalized to whole lung area (mm^2) at mock or SARS-CoV-2 MA10-infected 1-year-old female BALB/c mice at indicated time points. Graphs represent individuals necropsied at each timepoint with the average value for each treatment and error bars representing standard error of the mean. Wilcoxon rank-sum test was used to test the difference in CD4$^+$ or CD8$^+$ T cells identified by immunohistochemistry between two groups **(C, D)**. **(E to H)** Quantification of cells by flow cytometry is shown, including **(E)** total live cell counts; **(F)** total CD326$^+$ epithelial, CD31$^+$ endothelial, and CD45$^+$ immune cells; **(G)** CD4$^+$, CD8$^+$, regulatory T cells (Tregs), and CD19$^+$ B cells; and **(H)** subsets of myeloid lineage immune cells. Alv, alveolar; Mac, macrophage; Mono, monocyte; DC, dendritic cell; Inflam, inflammatory. Graphs include individuals selected for flow cytometry with average and error bars representing the standard error of the mean. Mock individuals are represented by open black circles and SARS-CoV-2 infected are represented by closed red circles in (**E to H**). Flow cytometry data were analyzed by Wilcoxon rank-sum test (**E**) or ANOVA followed by Sidak's multiple comparisons test (**F to H**).

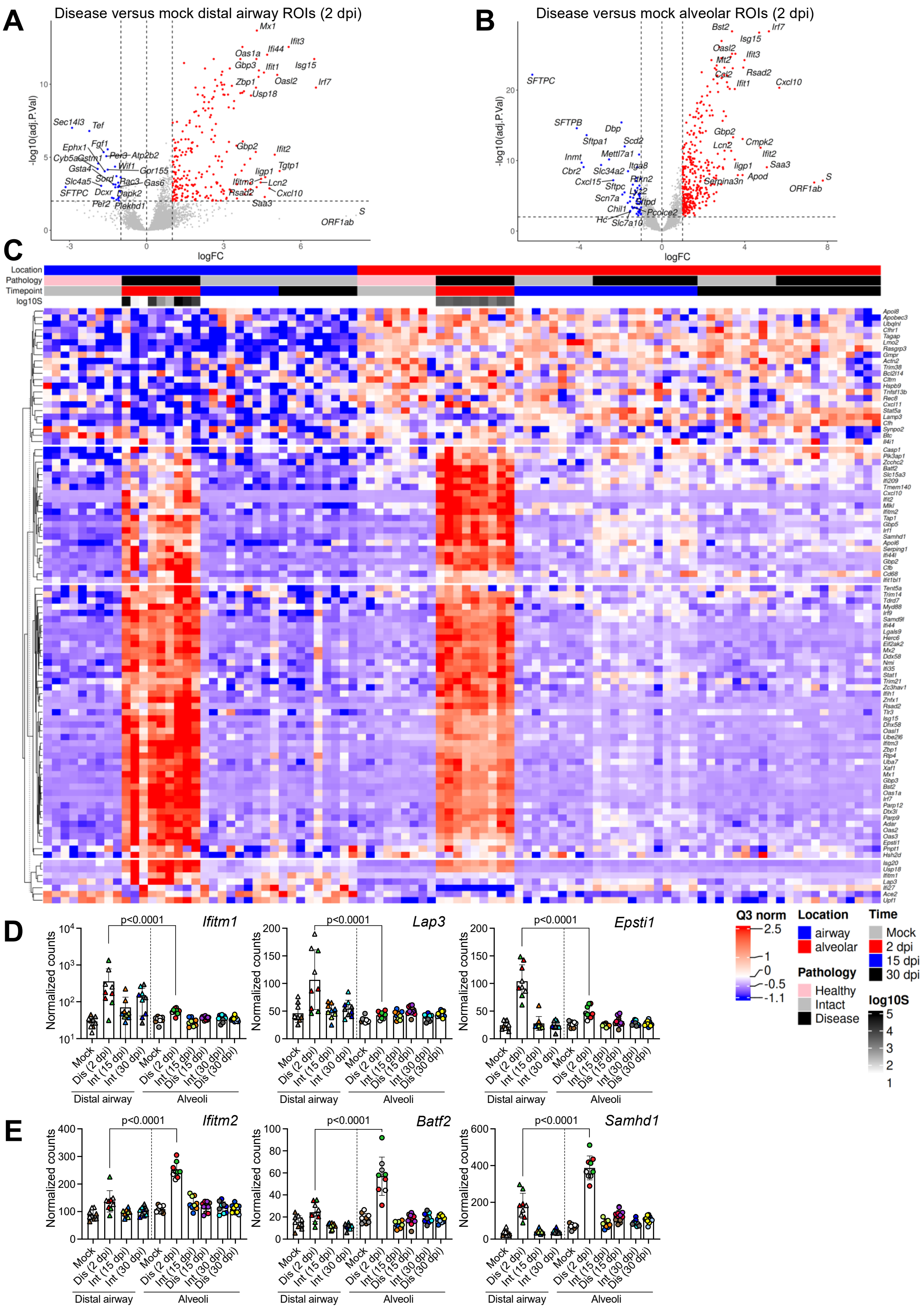
A
Disease versus mock distal airway ROIs (2 dpi)
-log10(adj.P.Val)
logFC
B
Disease versus mock alveolar ROIs (2 dpi)
-log10(adj.P.Val)
logFC
C
Location
Pathology
Timepoint
log10S
D
p<0.0001
Ifitm1
Lap3
Epsti1
Normalized counts
Mock
Dis (2 dpi)
Int (15 dpi)
Int (30 dpi)
Dis (15 dpi)
Dis (30 dpi)
Distal airway
Alveoli
E
Ifitm2
Batf2
Samhd1
Q3 norm
Location
airway
alveolar
Pathology
Healthy
Intact
Disease
Time
Mock
2 dpi
15 dpi
30 dpi
log10S

64

Fig. S6: Upregulation of interferon stimulated gene (ISG) expression after SARS-CoV-2 infection. (A and B) Volcano plots of digital spatial profiling (DSP) differentially expressed genes (DEGs) in SARS-CoV-2 MA10 infected **(A)** distal airway or **(B)** alveolar regions of interest (ROIs) at 2 dpi (D2) versus mock 1-year-old female BALB/c mice. **(C)** DSP heatmap of selected ISGs in alveolar and distal airway epithelial ROIs across all time points in mock and SARS-CoV-2 MA10-infected 1-year-old female BALB/c mice. DSP Q3 normalized counts of SARS-CoV-2 MA10 Spike (*S*) of ROIs are log10 transformed and represented by gray color intensity. **(D and E)** DSP Q3 normalized counts are shown for selected ISGs highly upregulated in **(D)** distal airway disease ROIs or **(E)** alveolar disease ROIs at 2 dpi in SARS-CoV-2 MA10-infected 1-year-old female BALB/c mice. Graphs represent all ROIs selected with triangles representing distal airway, circles representing alveoli, and each unique color representing one animal; bars represent average value of each group with error bars representing standard error of the mean in **(D and E)**. The difference in DSP Q3 normalized counts for targeted genes in ROIs between each condition and time point was statistically tested using a linear mixed-effect model with condition and time point as fixed effects and replicate mice as random-effect factors.

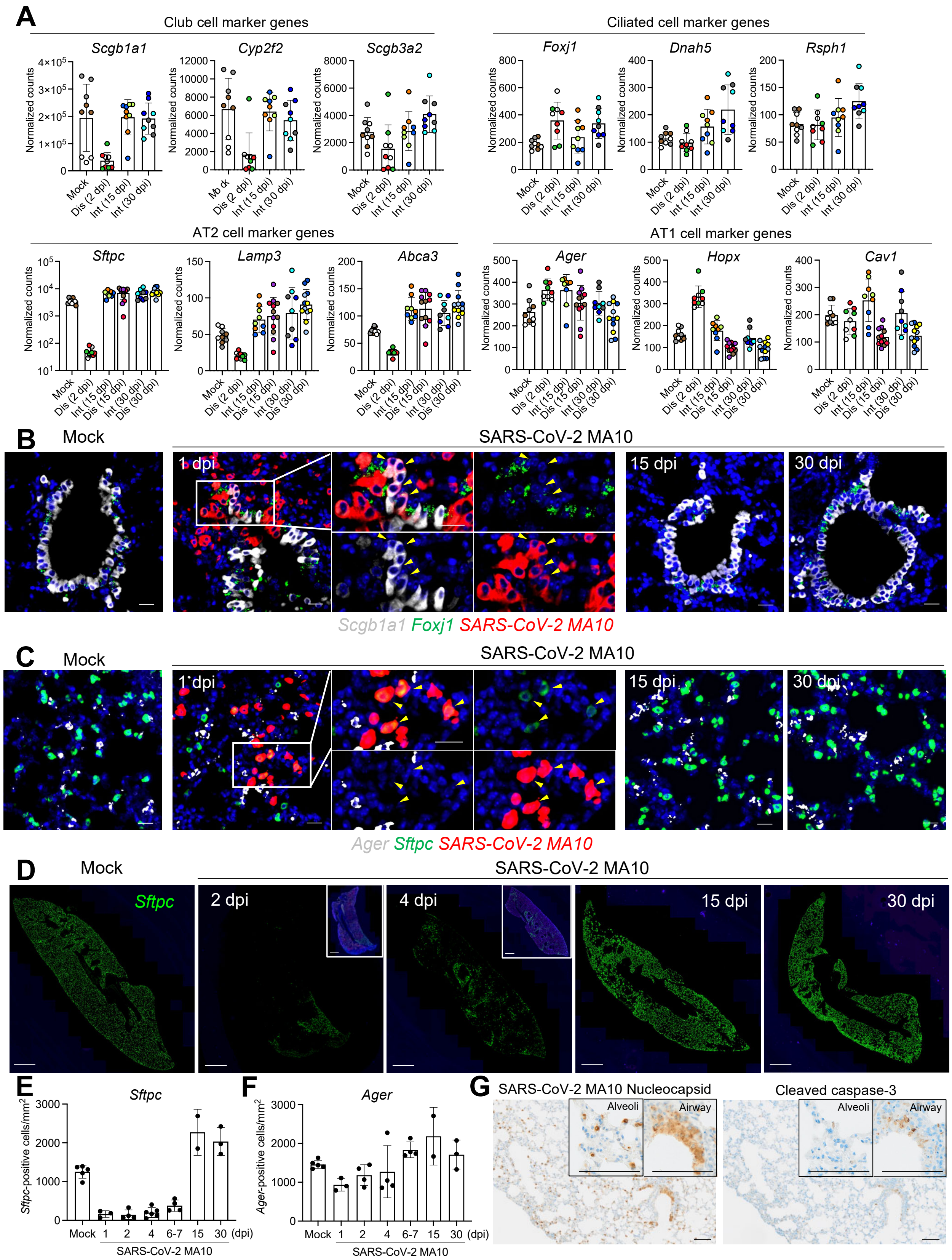

A
Club cell marker genes
Scgb1a1
Cyp2f2
Scgb3a2
Ciliated cell marker genes
Foxj1
Dnah5
Rsph1
Normalized counts
Mock
Dis (2 dpi)
Int (15 dpi)
Int (30 dpi)
AT2 cell marker genes
Sftpc
Lamp3
Abca3
AT1 cell marker genes
Ager
Hopx
Cav1
Dis (15 dpi)
Dis (30 dpi)
Int (30 dpi)
B
Mock
SARS-CoV-2 MA10
1 dpi
15 dpi
30 dpi
Scgb1a1 Foxj1 SARS-CoV-2 MA10
C
Mock
SARS-CoV-2 MA10
1 dpi
15 dpi
30 dpi
Ager Sftpc SARS-CoV-2 MA10
D
Mock
SARS-CoV-2 MA10
Sftpc
2 dpi
4 dpi
15 dpi
30 dpi
E
Sftpc
Sftpc-positive cells/mm²
Mock 1 2 4 6-7 15 30 (dpi)
SARS-CoV-2 MA10
F
Ager
Ager-positive cells/mm²
Mock 1 2 4 6-7 15 30 (dpi)
SARS-CoV-2 MA10
G
SARS-CoV-2 MA10 Nucleocapsid
Cleaved caspase-3
Alveoli
Airway

Fig. S7. SARS-CoV-2 MA10 infection causes transient loss of club and Alveolar Type II (AT2) cells. **(A)** DSP Q3 normalized counts for club (top left), ciliated (top right), AT2 (bottom left), and AT1 (bottom right**)** cell markers in mock, infected diseased (Dis), or intact (Int) distal airway (top row) or alveolar (bottom row) ROIs in mock and SARS-CoV-2 MA10-infected 1-year-old female BALB/c mice. Graphs represent all ROIs selected with each unique color representing one animal; bars represent average value of each group with error bars representing standard error of the mean. **(B)** RNA in situ hybridization (RNA-ISH) is shown for club (*Scgb1a1*) and ciliated (*FoxJ1*) markers at indicated timepoints in mock and SARS-CoV-2 MA10-infected 1-year-old female BALB/c mice. Arrow heads indicate colocalization of *SARS-CoV-MA10 RNA* with *Scgb1a1*. **(C)** RNA-ISH is shown for AT2 (*Sftpc*) and AT1 (*Ager*) markers at indicated timepoints in mock and SARS-CoV-2 MA10-infected 1-year-old female BALB/c mice. Arrowheads indicate colocalization of *SARS-CoV-MA10 RNA* with *Sftpc*. In **(B and C)**, scale bars = 20 μm. **(D)** Low magnification RNA-ISH is shown for *Sftpc* at indicated time points in mock and SARS-CoV-2 MA10-infected 1-year-old female BALB/c mice. Scale Bars = 1 mm. **(E and F)** Quantification of RNA-ISH is shown for **(E)** *Sftpc* or **(F)** *Ager* at indicated timepoints with average and standard error of the mean plotted. **(E)** *Sftpc* or **(F)** *Ager*-positive cells were counted and normalized to whole lung area (mm^2). **(G)** Immunohistochemistry for SARS-CoV-2 Nucleocapsid protein and cleaved caspase-3 is shown for lung samples collected at 2 dpi in SARS-CoV-2 MA10-infected 1-year-old female BALB/c mice. Scale Bars = 100 μm.

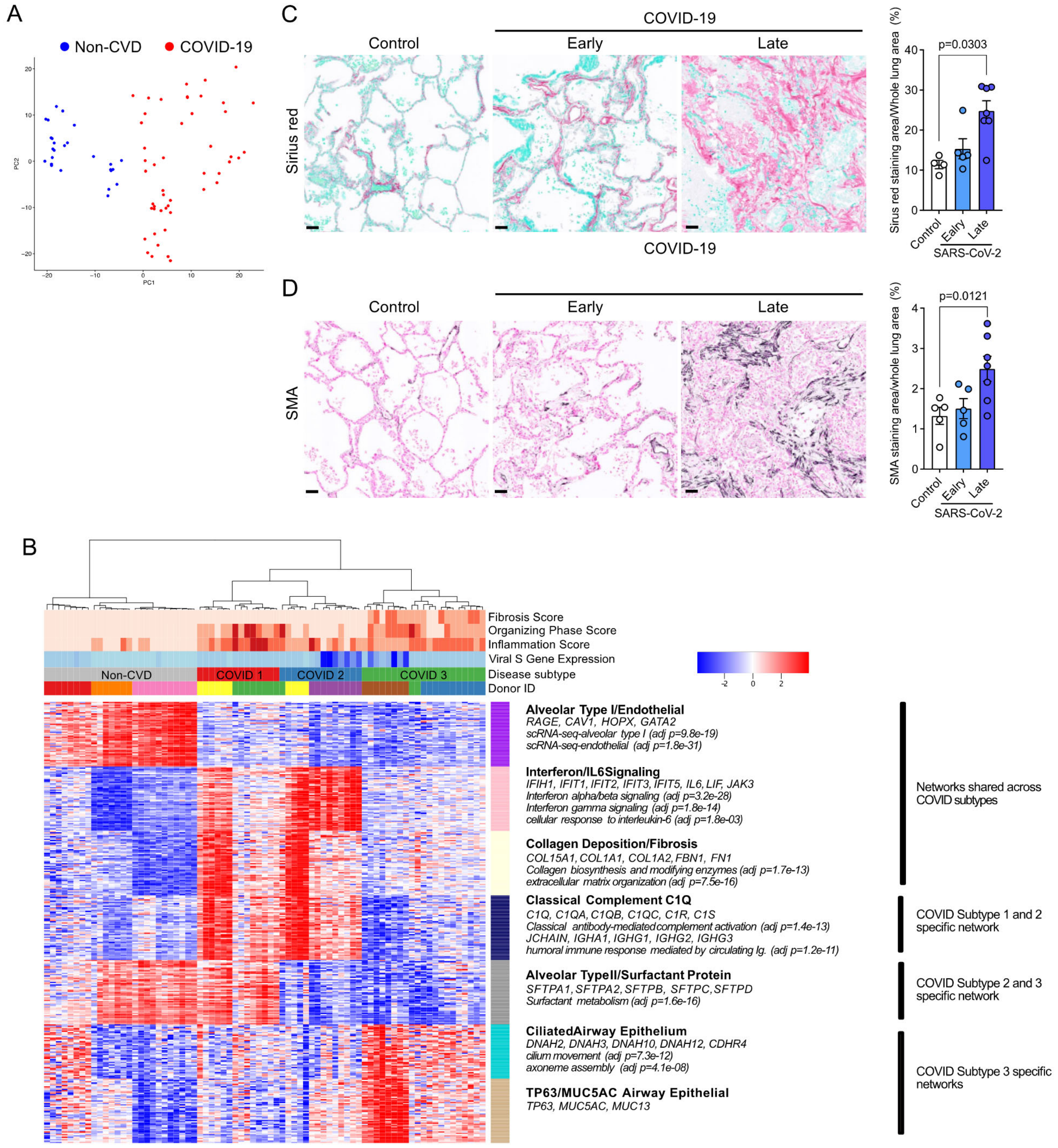

Fig. S8. SARS-CoV-2 MA10 pathogenesis closely resembles late human coronavirus disease 2019 (COVID-19). (A) Principal component analysis (PCA) plot of DSP alveolar ROIs selected in non-COVID-19 (non-CVD) (n=3) and COVID-19 (n=5) human donor lungs. **(B)** DSP heatmap of 7 identified networks in alveolar ROIs obtained from non-COVID-19 controls and individuals with COVID-19. Hierarchical clustering segregated COVID-19 ROIs into three subtypes (COVID 1, COVID 2, and COVID 3). DSP Q3

normalized counts of SARS-CoV-2 Spike (*S*) gene and histopathological scoring of alveolar ROIs for inflammation, organizing phase of lung injury, and fibrosis are shown. **(C and D)** Histopathological analysis of fibrotic features in control (n=4), early (n=5), and late (n=6) COVID-19 autopsy lungs by Picrosirius red (bright red-pink) for collagen deposition (**C**) and DAB/nickel-labeling (black) immunohistochemistry for smooth muscle actin (SMA) (**D**). Scale Bars = 50 μm. Picrosirius red and SMA immunohistochemistry quantification represents average value for each group, with error bars representing standard error of the mean. Wilcoxon rank-sum test was used to test the difference in normalized Picrosirius red- or SMA-stained areas identified by immunohistochemistry between two groups.

Supplemental Table 1: Reagents and Materials

Material	Company/Source	Product/Reference Number
**Antibodies**		
SARS-CoV-1 nucleocapsid, dilution at 1:8000	Novus Biologicals	Cat#NB100-56576
Rabbit polyclonal SARS coronavirus nucleocapsid, dilution at 1:500	Invitrogen	Cat#RRID: AB_1087200
Goat anti-CCSP, dilution at 1:3000	Sigma-Aldrich	Cat#ABS1673
Rat DC-LAMP (LAMP3) antibody, dilution at 1:100	NOVUS Biologicals	Cat#1010E1.01
Alpha Smooth Muscle Actin Antibody [1:2000]	Abcam	Cat#ab124964
Picrosirius Red	Polysciences Inc.	Cat#24901-500
Hematoxylin	Richard Allen Scientific	Cat#7231L
CD4 Antibody [1:1000]	Abcam	Cat#ab183685
CD8 Antibody [1:100]	Invitrogen	Cat#14-0808-82
B220 Antibody [1:500]	BD Pharmingen	Cat#550286
Ly6G Antibody [1:500]	Abcam	Cat#ab238132
CD68 Antibody 1:100	Abcam	Cat#ab125212
iNos Antibody [1:175]	Millipore	Cat#ABN26
Arginase Antibody [1:100]	Cell Signaling	Cat#93668s
Alpha Smooth Muscle Actin Antibody [1:2000]	Abcam	Cat#ab124964
CD45 Antibody (GeoMx Solid Tumor TME Morphology Kit-Mouse)	NanoString	Cat#121300304
COL1A1 Antibody, dilution at 1:500	Abcam	Cat#ab21286
Human SMA Antibody [1:1000]	Abcam	Cat#ab5694
Sftpc Antibody [1:300]	Millipore	Cat#AB3786
Krt8 Antibody [1:50]	DSHB	Cat#TROMA-I
Ager Antibody [1:300]	R&D	Cat#AF1145
GeoMx Solid Tumor TME Morphology Kit-Human	NanoString	Cat#121300310
CD103 BV786 (clone M290)	BD Biosciences	Cat#564322
FcgRIII/FcgRII (clone 2.4G2)	BD Biosciences	Cat#553141
Ki67 PE (clone B56)	BD Biosciences	Cat#556027
Siglec F PE (clone E50-2440)	BD Biosciences	Cat#552126
CD103 PE/Cy7 (clone 2E7)	BioLegend	Cat#121426
CD104 APC (clone 346-11A)	BioLegend	Cat#123612
CD104 FITC (clone 346-11A)	BioLegend	Cat#123606
CD11b APC Cy7 (clone M1/70)	BioLegend	Cat#101216
CD11c BV 605 (clone N418)	BioLegend	Cat#117334
CD14 PE-Cy7 (clone Sa14-2)	BioLegend	Cat#123316
CD19 BV605 (clone 6D5)	BioLegend	Cat#115540
CD24 PerCP-Cy5.5 (clone M1/69)	BioLegend	Cat#101824
CD3 FITC (clone 145-2C11)	BioLegend	Cat#100306
CD31 PB (BV421) (clone 390)	BioLegend	Cat#102422
CD326 PE-Dazzle (clone G8.8)	BioLegend	Cat#118236
CD4 Alexa 700 (clone GK1.5)	BioLegend	Cat#100429
CD45 BV785 (clone 30-F11)	BioLegend	Cat#103149
CD45 FITC (clone 30-F11)	BioLegend	Cat#103108
CD64 PE-Cy7 (clone X54-5/7.1)	BioLegend	Cat#139314
CD8a PE-CF594 (clone 53.6.7)	BioLegend	Cat#100762
Foxp3 Alexa 647 (clone 150D)	BioLegend	Cat#320014
g/d PerCP-Cy5.5 (clone GL3)	BioLegend	Cat#118108

Ly6C PB (clone HK1.4)	BioLegend	Cat#128024
Ly6G APC (clone 1A8)	BioLegend	Cat#127614
MHCII Alexa 700 (clone M5/114.15.2)	BioLegend	Cat#107618
NK-1.1 BV785 (clone PK136)	BioLegend	Cat#108749
Streptavidin BV421	BioLegend	Cat#405225
Streptavidin BV605	BioLegend	Cat#405229
T1 alpha biotin (clone 8.1.1)	BioLegend	Cat#127404
Zombie aqua	BioLegend	Cat#423102
Zombie NIR	BioLegend	Cat#423106
Donkey anti-Rabbit IgG (H+L) Highly Cross-Adsorbed Secondary Antibody, Alexa Fluor 555, dilution at 1:1000	Invitrogen	Cat#AB_162543
Donkey anti-Rat IgG (H+L) Highly Cross-Adsorbed Secondary Antibody, Alexa Fluor 488, dilution at 1:1000	Invitrogen	Cat#AB_2535794
Donkey anti-Goat IgG (H+L) Cross-Adsorbed Secondary Antibody, Alexa Fluor 647, dilution at 1:1000	Thermo Fisher Scientific	Cat#AB_2535864
Discovery OmniMap anti Rabbit HRP	Ventana, Roche	Cat#760-4311
**Bacterial and Virus Strains**		
SARS-CoV-2 MA10	Leist *et al.*, 2020	GenBank: MT952602
**Chemicals, Peptides, and Recombinant Proteins**		
TRIzol Reagent	Thermo Fisher Scientific	Cat#15596026
**Critical Commercial Assays**		
Immune Monitoring 48-Plex Mouse ProcartaPlex Panel	Invitrogen	EPX480-20834-901
GeoMx DSP Collection Plate	NanoString	100437
GeoMx Instrument Buffer Kit PCLN	NanoString	100474
GeoMx RNA Slide Prep FFPE PCLN	NanoString	121300313
GeoMx Seq Code Pack_AB	NanoString	121400201
GeoMx NGS RNA WTA Mm	NanoString	121401103
GeoMx Nuclear Stain Morphology Kit	NanoString	121300303
GeoMx COV19 Imm Resp Atlas	NanoString	121300314
RNAScope Multiplex Fluorescent Reagent Kit v2	ACD	Cat#323100
RNAScope probe SARS-CoV-2, S gene encoding the spike protein (channel 1, 2, and 3)	ACD	Cat#848561
RNAScope probe Ager (channel 1)	ACD	Cat#550791
RNAScope probe Sftpc (channel 2)	ACD	Cat#314101-C2
RNAScope probe Foxj1 (channel 2)	ACD	Cat#317091-C2
RNAScope probe Scgb1a1 (channel 3)	ACD	Cat#420351-C3
RNAScope probe Fn1	ACD	Cat#316951
RNAScope probe Spp1	ACD	Cat#435191
RNAScope probe C3	ACD	Cat#417841
RNAScope probe Tgfb1	ACD	Cat#407751
RNAScope probe Krt8 (channel 3)	ACD	Cat#424521-C3
RNAScope probe Cdkn1a (channel 2)	ACD	Cat#408551-C2
RNAScope probe SARS-CoV-2, S gene encoding the spike protein (channel 3)	ACD	Cat#848568

Vector TrueVIEW Autofluorescence Quenching Kit	Vector Laboratories	Cat#SP-8400
**Experimental Models: Cell Lines**		
Vero E6	ATCC	Cat#CRL1586
**Experimental Models: Organisms/Strains**		
Mouse: BALB/c: BALB/cAnNHsd	Envigo	Strain 047
Mouse: C57Bl/6J	The Jackson Labs	Strain 000664
**Software and Algorithms**		
FinePointe (Version 2.3.1.16)	DSI Buxco respiratory solutions, DSI Inc.	https://www.datasci.com/products/software/finepointe-software
xPONENT (Version 4.3)	Luminex	https://www.luminexcorp.com/xponent/
GraphPad Prism (Version 8.4.3)	GraphPad	https://graphpad.com
Adobe Illustrator (Version 24.2.3)	Adobe	https://www.adobe.com/products/illustrator.html
Visiopharm	Visiopharm	https://visiopharm.com/
Olyvia (Version 3.1.1)	Olympus	https://olympus-lifescience.com
**Equipment**		
Whole body plethysmography machine	DSI Buxco respiratory solutions, DSI Inc.	N/A
GeoMx	NanoString	N/A
MAGPIX machine	Luminex	N/A
Leica ASP 6025	Leica	N/A
Leica Paraplast	Leica	N/A
Ventana Discovery platform	Roche	N/A
Olympus BX43 light microscope/ Olympus DP27 camera	Olympus	N/A

Supplemental Table 2: Demographics of donors used for GeoMx DSP.

Age	Sex	Health Status	RNA-ISH SARS-CoV-2	Days Intubated
76	F	Non-COVID-19	Negative	
24	F	Non-COVID-19	Negative	
42	F	Non-COVID-19	Negative	
57	M	COVID-19	Negative	21
67	M	COVID-19	Negative	17
77	M	COVID-19	Positive	NI
57	F	COVID-19	Positive	DNI
79	F	COVID-19	Positive	DNI

DNI: Do Not Intubation, NI: No intubation (Cardiac arrest before intubation)

Supplemental Table 3: Demographics of donors used for quantification of Sirius Red and IHC SMA staining.

Age	Sex	Health Status	Days Intubated	Days of Disease	COVID-19 Phase*
43	M	Non-COVID-19			
27	M	Non-COVID-19			
48	M	Non-COVID-19			
37	M	Non-COVID-19			
24	M	Non-COVID-19			
40	M	COVID-19	0	3	Early
91	F	COVID-19	0	1	Early
77	M	COVID-19	0	6	Early
42	M	COVID-19	0	0	Early
92	M	COVID-19	0	11	Early
42	M	COVID-19	Unknown	30	Late
58	M	COVID-19	0	23	Late
69	M	COVID-19	17	32	Late
30	F	COVID-19	25	33	Late
58	M	COVID-19	13	49	Late
61	M	COVID-19	32	36	Late
52	M	COVID-19	29	35	Late

*Early- and late- phase specimens were defined as autopsy tissue obtained ≤ 20 and > 20 days post the onset of symptoms, respectively.

Supplementary Data Files:

Data File 1: Raw data matrix for cytokine data shown in heatmap in fig. S4.

Data File 2: Gene lists and clustering information for all heatmaps included in this manuscript.

Data File 3: Raw and normalized read counts for mouse digital spatial profiling (DSP) data.

Data File 4: Differential gene expression (DEG) analysis of mouse DSP data.

Data File 5: Reactome pathway analysis of mouse DSP data.

Data File 6: Raw and normalized read counts for human DSP data.

Data File 7: Individual-level raw data for all graphs included in this manuscript.
